# The global atlas of edible insects: analysis of diversity and commonality contributing to food systems and sustainability

**DOI:** 10.1038/s41598-024-55603-7

**Published:** 2024-02-29

**Authors:** Evanson R. Omuse, Henri E. Z. Tonnang, Abdullahi Ahmed Yusuf, Honest Machekano, James Peter Egonyu, Emily Kimathi, Samira Faris Mohamed, Menale Kassie, Sevgan Subramanian, Juliet Onditi, Serah Mwangi, Sunday Ekesi, Saliou Niassy

**Affiliations:** 1 International Centre of Insect Physiology and Ecology (icipe), P.O. Box 30772-00100, Nairobi, Kenya; 2 Department of Zoology and Entomology, University of Pretoria, Private Bag X20, Hatfield, 0028 South Africa; 3 Faculty of Science and Education, Busitema University, Tororo, Uganda; 4 Inter-African Phytosanitary Council of African Union (AU-IAPSC), P.O Box 4170, Yaoundé, Cameroon

**Keywords:** Ecology, Climate sciences, Ecology

## Abstract

The future of the food system on the planet is increasingly facing uncertainties that are attributable to population growth and a surge in demand for nutritious food. Traditional agricultural practices are poised to place strain on production, as well as natural resources and ecosystem services provided, particularly under a changing climate. Given their remarkable attributes, including a low environmental footprint, high food conversion ratio, rapid growth and nutritional values, edible insects can play a vital role in the global food system. Nonetheless, substantial knowledge gaps persist regarding their diversity, global distribution, and shared characteristics across regions, potentially impeding effective scaling and access to edible insects. Therefore, we compiled and analysed the fragmented database on edible insects and identified potential drivers that elucidate insect consumption, globally, focusing on promoting a sustainable food system. We collated data from various sources, including the literature for a list of edible insect species, the Global Biodiversity Information Facility and iNaturalist for the geographical presence of edible insects, the Copernicus Land Service library for Global Land Cover, and FAOSTAT for population, income, and nutritional security parameters. Subsequently, we performed a series of analytics at the country, regional and continental levels. Our study identifies 2205 insect species, consumed across 128 countries globally. Among continents, Asia has the highest number of edible insects (932 species), followed by North America (mainly Mexico) and Africa. The countries with the highest consumption of insects are Mexico (450 species), Thailand (272 species), India (262 species), DRC (255 species), China (235 species), Brazil (140 species), Japan (123 species), and Cameroon (100 species). Our study also revealed some common and specific practices related to edible insect access and utilisation among countries and regions. Although insect consumption is often rooted in cultural practices, it exhibits correlations with land cover, the geographical presence of potentially edible insects, the size of a country’s population, and income levels. The practice of eating insects is linked to the culture of people in Africa, Asia, and Latin America, while increased consciousness and the need for food sustainability are driving most of the European countries to evaluate eating insects. Therefore, edible insects are becoming an increasingly significant part of the future of planetary food systems. Therefore, more proactive efforts are required to promote them for their effective contribution to achieving sustainable food production.

## Introduction

Food security has emerged as a central global concern for human existence and continues raising questions about various dimensions of food systems, including economic, social, cultural, and political aspects^1^. By 2050, the global population is projected to reach 9.8 billion people^2^, and our food demand is expected to increase from 59 to 98%^3^. This substantial growth in food demand will place immense pressure on natural resources, such as water and land, raising concerns about the sustainability of current agricultural production systems. Climate change-induced challenges, such as soil erosion, erratic rainfall, and invasive pests, will further strain agricultural productivity. Further, global food system carbon emissions could preclude reaching climate change targets of restricting temperature increases to 1.5 °C and 2 °C^4^. Consequently, there is an urgent need to develop new sustainable but equitable food systems, accompanied by the appropriate policies to ensure the good health and prosperity of people, worldwide^1,5^.

Protein remains a critical driver of the planetary food systems^6^. The demand for meat has tripled over the past 50 years, and currently, the global annual production stands at about 340 million metric tons, equivalent to about 80 billion animals slaughtered^7^. Annual per capita meat consumption is estimated at a remarkable average of 43 kg^7^. Accompanying this is the demand for animal feeds, which rely on protein sources such as fish meal and soybean, which compete with human protein needs, thus stressing the scarce water and land resources. The production and supply of animal feed depend heavily on fuel costs and the global geopolitical situation, which dictate their price, availability, and access^8^. In 2018, an estimated 1.1 billion tons of animal feed was produced^9^.

Africa and Asia face significant deficits in essential food intake, particularly protein^10^. In 2022, the daily protein intake per capita was relatively low in Africa (64.4 g) followed by Asia (81.8 g), South America (87.8 g), Oceania (94.5 g), with the highest level being in North America (115.3 g) and Europe (104.1 g). Animal-derived foods (fish, meat, and eggs) accounted for the protein intake in these regions. Fat intake followed a similar pattern, with the lowest levels being in Africa (54.0 g) and Asia (79.5 g), compared with North America (179.5 g) and Europe (136.2 g). However, out of more than 6000 food crops, only 9 crops (maize, rice, wheat, potatoes, soybeans, cassava, sugar cane, oil-palm fruit, and sugar beet) contribute more than 66% of food consumed^11^. These disparities underscore the need for a paradigm shift to be made in the global food system to achieve the United Nations Sustainable Development Goal 2 (No Hunger), especially in Africa, where protein and fat intake remain low.

Edible insects have generated attention as a sustainable protein source, with crude protein content ranging from 40 to 75^12–15^, surpassing that of animal protein (12% to 34.5%)^16^ and plant proteins (7% to 50%)^17^. Insects are highly edible, with about 80% of their body weight being consumable, compared with 55% for chickens and pork, and 40% for cattle^18^. Edible insects also offer diverse essential nutrients, antimicrobials, enzymes, and chitin^12,15,19^.

Furthermore, insects can be reared or domesticated in numbers as, what is commonly called, “minilivestock”^20^. However, only 6% of edible insect species have been successfully reared, while 94% are still gathered in the wild^21^. Insect feed conversion ratios are significantly more efficient than those of traditional livestock are. For instance, crickets need twelve times less feed than cattle do, four times less than sheep do, and twice less than pigs and broiler chickens do, to produce the same amount of protein^22^. In terms of resource efficiency, 1 kg of beef requires considerably more water (3000 times), feed (12.5 times), and significant greater areas for husbandry than what is required to produce 1 kg of crickets^14,23,24^.

Furthermore, some insects are best suited for a sustainable circular economy. For instance, the black soldier fly (BSF) *Hermetia illucens* L. (Diptera: Stratiomyidae) can convert waste into organic fertiliser that feeds back into agriculture, while their larvae serve as a protein source for animal feed^25^. They can be mass-reared on extremely low-cost waste materials, thereby doubling, and diversifying the benefits of entomophagy, i.e. low-cost, high-value protein, waste recycling, soil, and environmental health, amongst other benefits. The use of BSF for waste recycling is timely, as it is estimated that, globally, around two billion tons of solid waste (at least 50% of food and green waste) is produced annually, but only 33% is recycled efficiently^26^. The need for food-producing technologies that require limited resources (land, water and labour), and with little environmental impact, highlights the growing relevance of edible insects in a circular economy and regenerative agriculture for securing the sustainability of food systems on our planet^27,28^. Insects will leave a lighter negative environmental footprint, such as in greenhouse gas emissions, than conventional livestock production does^13,27,29^. Despite the potential multifaceted benefits of edible insects for humankind, global economies and the environment, limited efforts have been deployed to collate facts on their global diversity, similarities in their access and consumption, and contribution to the sustainable food system on the continent.

Over 1900 insect species are considered as forming part of the traditional diets of at least two billion people^13,30–32^, representing approximately 30% of the world’s human population. It is estimated that people in 113 countries consume at least one insect species^33^, and Africa, Latin America, and Asia have a well-established history of insect consumption^12^. Increasing advocacy for, and lobbying by, diverse UN agencies, governments and non-governmental organisations have rapidly increased not only awareness, but also consciousness, of the benefits of insects as food and feed across the globe. Despite the awareness, the edible insect’s global diversity datasets remain scant and highly fragmented, although there have been a few attempts to consolidate and analyse these pieces of information and compare inter-country and inter-continental commonalities and specificities.

One of the principal explanations for insect consumption is that insects represent half of all living organisms; they are found in various habitats, such as forests, agricultural fields, and even aquatic ecosystems, and most of them depend on plants to survive. They represented the cheapest and primary source of animal protein during the earliest stages of humankind before the invention of agriculture. We therefore hypothesised that there could be a strong linkage between land cover, plant diversity and climate, and the diversity of edible insects. Yet, this has not been demonstrated. However, most recently, some edible insect species were shown to be on the verge of extinction^34^. Over-exploitation, unsustainable harvesting methods, and damage to habitats caused through deforestation, forest degradation and pollution (e.g. through excessive use of insecticides) are part of the drivers for the decline in abundance and diversity of edible insects^12,35,36^. In addition to anthropogenic factors, climate change is projected to affect the distribution and abundance of edible insects in ways that are still relatively unknown^12^. The regulatory framework governing food and feed chains, including the harvesting, rearing, processing, and marketing of insects, is in its infancy in most parts of the world^23,37^.

All over the world, wild harvesting, rearing, processing, and selling of edible insects across genders holds bright prospects for the planetary food system^12^. Indeed, the sustainable wild harvesting and farming of insects could offer diverse employment opportunities and much-needed cash income at the household level, and could support industrial development^12,38^. Initiatives to integrate and/or incorporate the ingredients of edible insects into conventional foods seem to be more appealing to consumers and significantly reduce rejection by communities that were not traditionally “entomophagous”^39,40^. Previous studies had projected that the edible insect market would grow from approximately USD 400 million in 2018 to USD 1.2 billion in 2023, signifying a global increase of roughly 25% annually, with Asia–Pacific and Latin America accounting for over 50% of the market, while North America and Europe would show a significant increase, compared with Africa^41,42^. Studies have also predicted that this global market might be worth around USD 8 billion by 2030. This was attributed to increasing awareness of the benefits of edible insects and strategic investment initiatives for using insects in food, feed, cosmetics, organic fertiliser, and pharmaceutical industries.

Despite the enormous benefits of edible insects, the stigma associated with them as “food for the poor” might result in outright rejection by some people. Without an in-depth understanding of edible insects in terms of their association with nutrition and health indices, gender-disaggregated income contribution, population growth and development, and Gross Domestic Product (GDP), the promotion of edible insects and their conservation will remain a formidable task. Insights into the global diversity and distribution of edible insects across countries and continents become pertinent in the advent of greater awareness and uptake for sustainable food systems. Providing validated information on the diversity and abundance of edible species at national, regional, and global levels might help to push these precious and sustainable natural food resources higher on political, investment and research agendas, worldwide.

This paper aims to analyse the global biodiversity of edible insects to guide policies on their conservation and domestication as sustainable food and feed sources. It describes factors influencing insect distribution, commonalities at regional and national levels, and the contribution of insects to the planetary food systems.

## Results

### Orders and families of edible insects in the world

**Figure 1:**
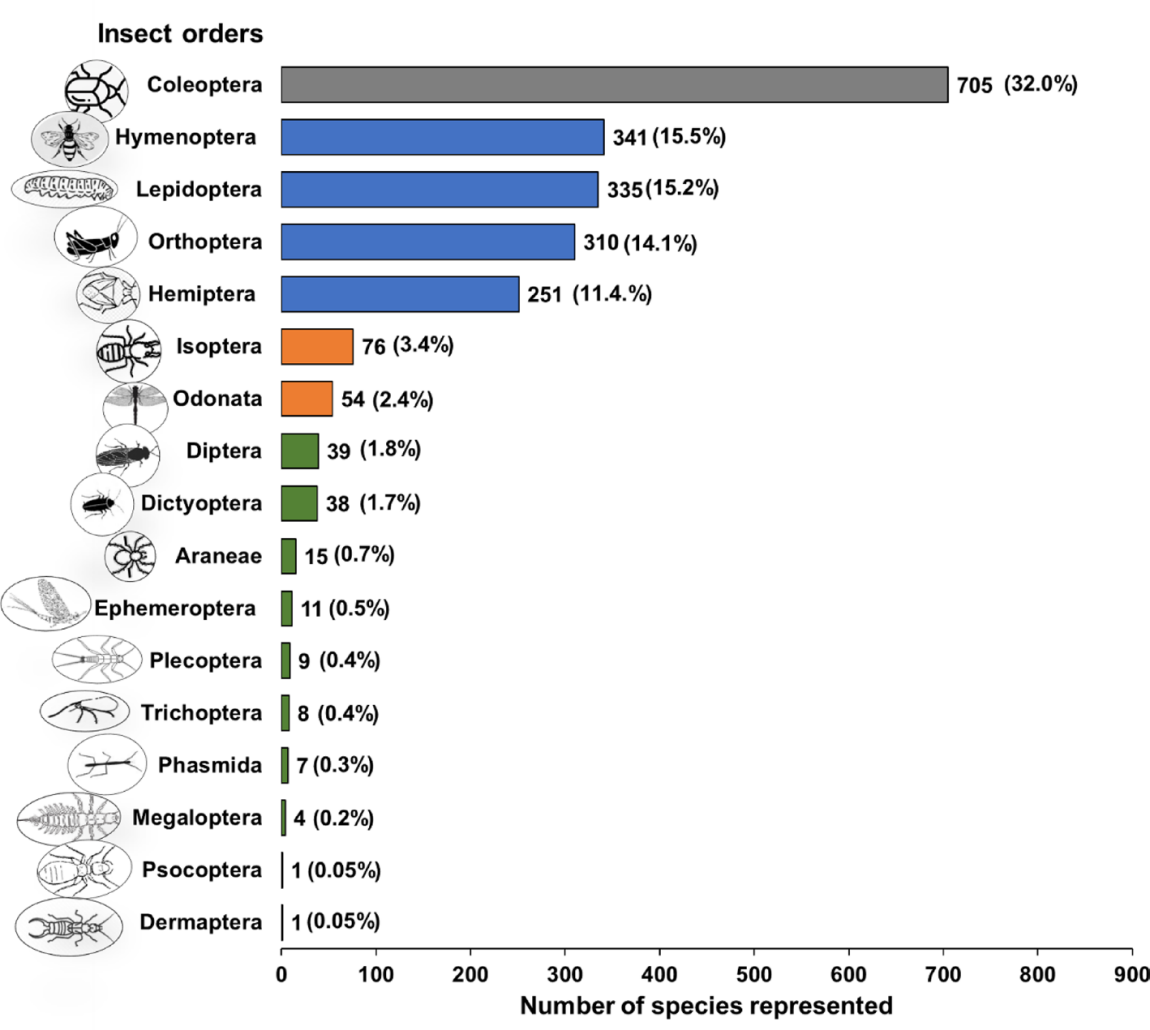
Global distribution of edible insect species by taxonomic order. Grey represents ≥ 500 species, Blue = 100–499 species, orange = 50–99 species and green 1–49 species.

Our comprehensive investigation identified 2205 insect species, from 25 distinct orders, that are confirmed as being edible (Fig. 1). These orders vary in their global dominance among the edible insect species. Coleoptera comprise the most prevalent order, constituting 32.0% of the edible insect species, followed by Hymenoptera (15.5%), Lepidoptera (15.2%), Orthoptera (14.1%), Hemiptera (11.4%), and several other orders with varying degrees of representation.

**Figure 2:**
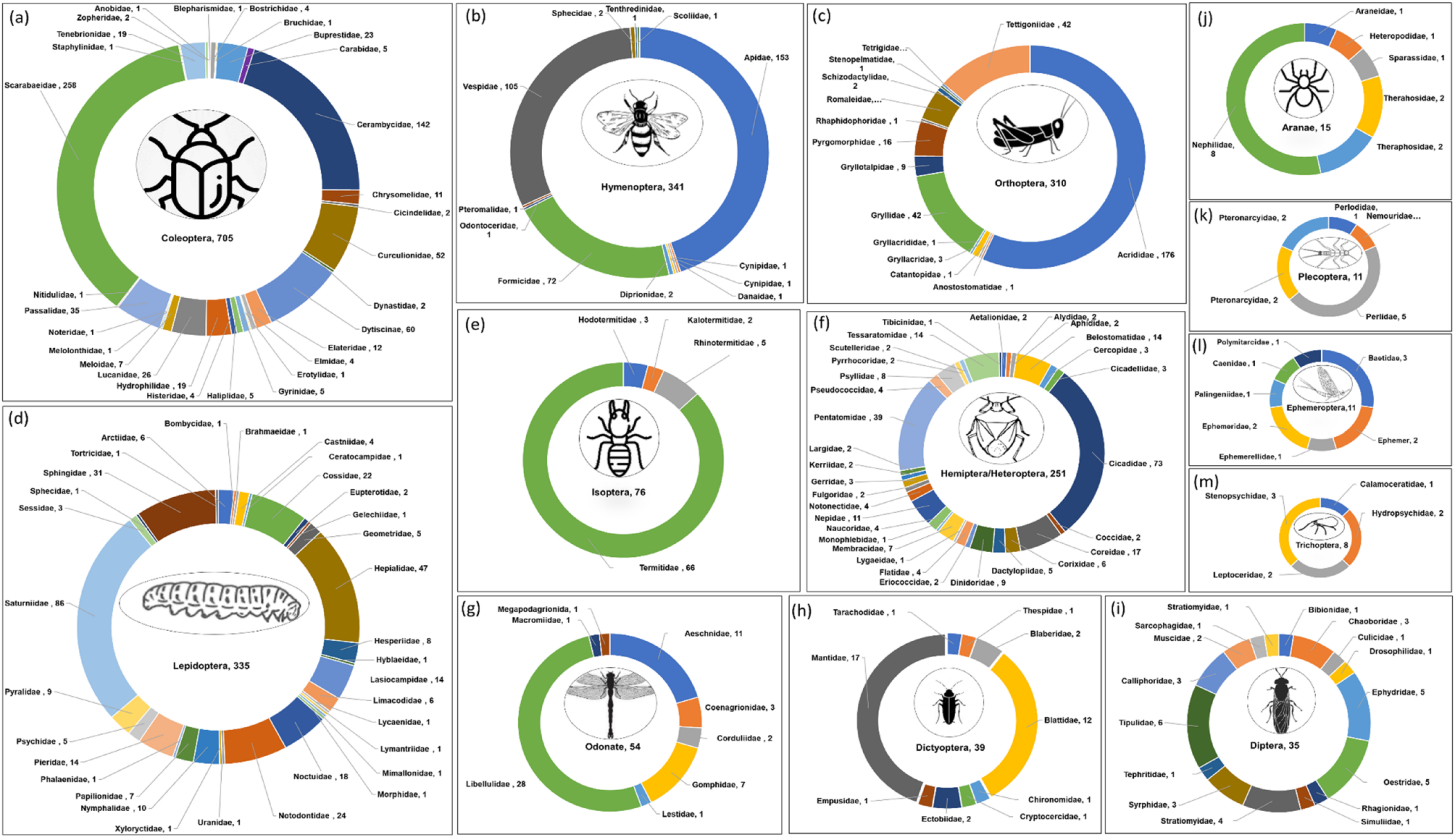
The number of edible insect species represented by their family and order. Insect orders: Coleoptera (**a**), Hymenoptera (**b**), Orthoptera (**c**), Lepidoptera (**d**), Isoptera (**e**), Hemiptera (**f**), Odonata (**g**), Dictyoptera (**h**), Diptera (**i**), Araneae (**j**), Plecoptera (**k**), Ephemeroptera (**l**), Trichoptera (**m**). The orders with edible insects belonging to only one family and few species, such as Dermaptera (Forficulidae), Megaloptera (Corydalidae), Phasmida (Phasmatidae), and Psocoptera (Psocidae), are not included in the figure.

The distribution of edible insect species, across orders and families, is presented in Fig. 2, revealing fascinating insights into the diversity of consumption. Within the orders Coleoptera, nine predominant families stand out, with over 10 species each: Scarabaeidae, Cerambycidae, Dytiscinae, Curculionidae, Passalidae, Lucanidae, Buprestidae, Hydrophilidae, Tenebrionidae, Elateridae and Chrysomelidae (Fig. 2a). The order Heteroptera boasts the family Cicadidae, Pentatomidae, Coreidae, Belostomatidae, Tessaratomidae and Nepidae as being the most consumed (Fig. 2f). The order Hymenoptera features Apidae, Formicidae and Vespidae (Fig. 2b). Termitidae comprise one family with many species (over 66) in the order Isoptera (Fig. 2e). Lepidoptera encompass several well-consumed families, such as Saturniidae, Hepialidae, Sphingidae, Cossidae, and Noctuidae (Fig. 2d). Odonata present Libellulidae and Aeschnidae as notable families (Fig. 2g), while Orthoptera boasts Acrididae, Tettigoniidae, Gryllidae and Romaleidae (Fig. 2c). Several orders are represented by a family, including Dermaptera (Forficulidae, 1), Megaloptera (Corydalidae, 4), Phasmida (Phasmatidae, 4), and Psocoptera (Psocidae, 1), showcasing the diverse array of edible insects across various taxonomic groups.

### Order and families of edible insects across continents

**Figure 3:**
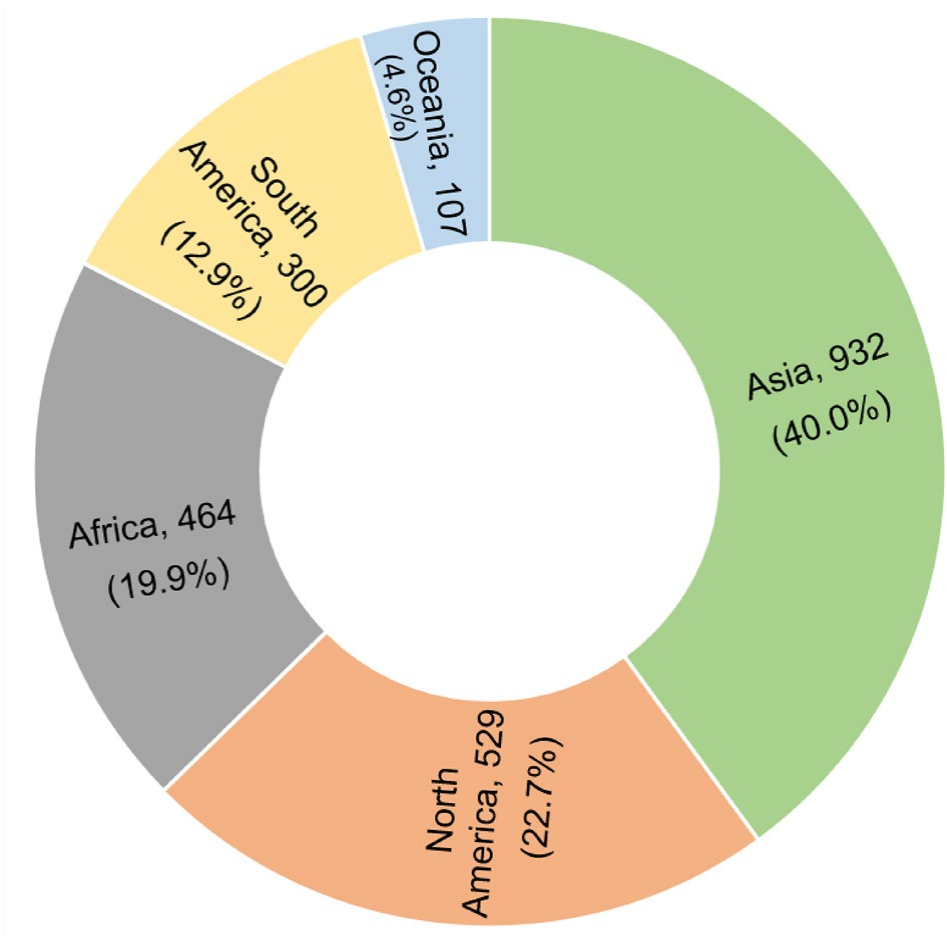
The number of edible insect species consumed in North and South America, Africa, Asia, and Oceania. Provided in brackets are the proportion of edible insects.

Asia has the largest number of edible insect species (932), followed by North America (including Mexico) (529), Africa (464), and South America (300). In contrast, Oceania has the least number of edible insects (107) (Fig. 3).

**Figure 4:**
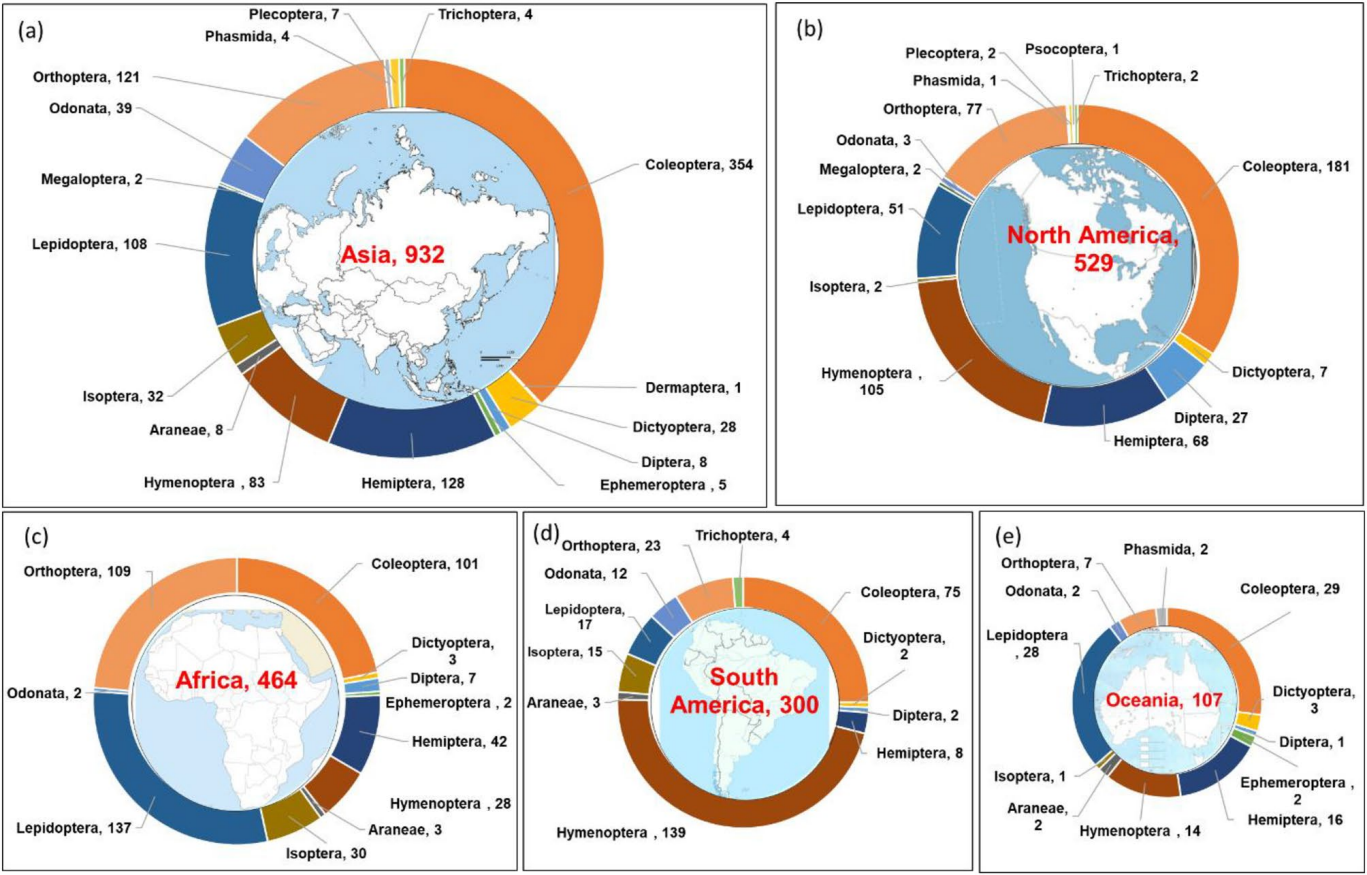
The number of edible insects reported from each continent is represented by insect orders and the number of species. Europe has been excluded because insects are not traditionally part of people’s diets.

The diversity of edible insect species across the five continents exhibited variations by insect order, as well as the number of orders present in each continent, as depicted in Fig. 4. In Asia, the top five orders in terms of consumption were Coleoptera (354), Hemiptera (128), Orthoptera (121), Lepidoptera (108), and Hymenoptera (83). North America’s prominent orders included Coleoptera (181), Hymenoptera (105), Orthoptera (77), Hemiptera (68), and Lepidoptera (51). In Africa, Lepidoptera (137), Orthoptera (109), Coleoptera (101), Hemiptera (42), and Isoptera (30) were the most frequently consumed orders. South America featured Hymenoptera (139), Coleoptera (75), Orthoptera (23), Lepidoptera (17), and Isoptera (15) as its predominant orders. Consumption pattern in Oceania region was characterised by Coleoptera (29), Lepidoptera (28), Hemiptera (16), Hymenoptera (14), and Orthoptera (7). These findings underscore the regional variations in edible insect preferences and highlight the significance of insect orders in shaping dietary choices across continents.

### Number of species of edible insects in each country across continents

**Figure 5:**
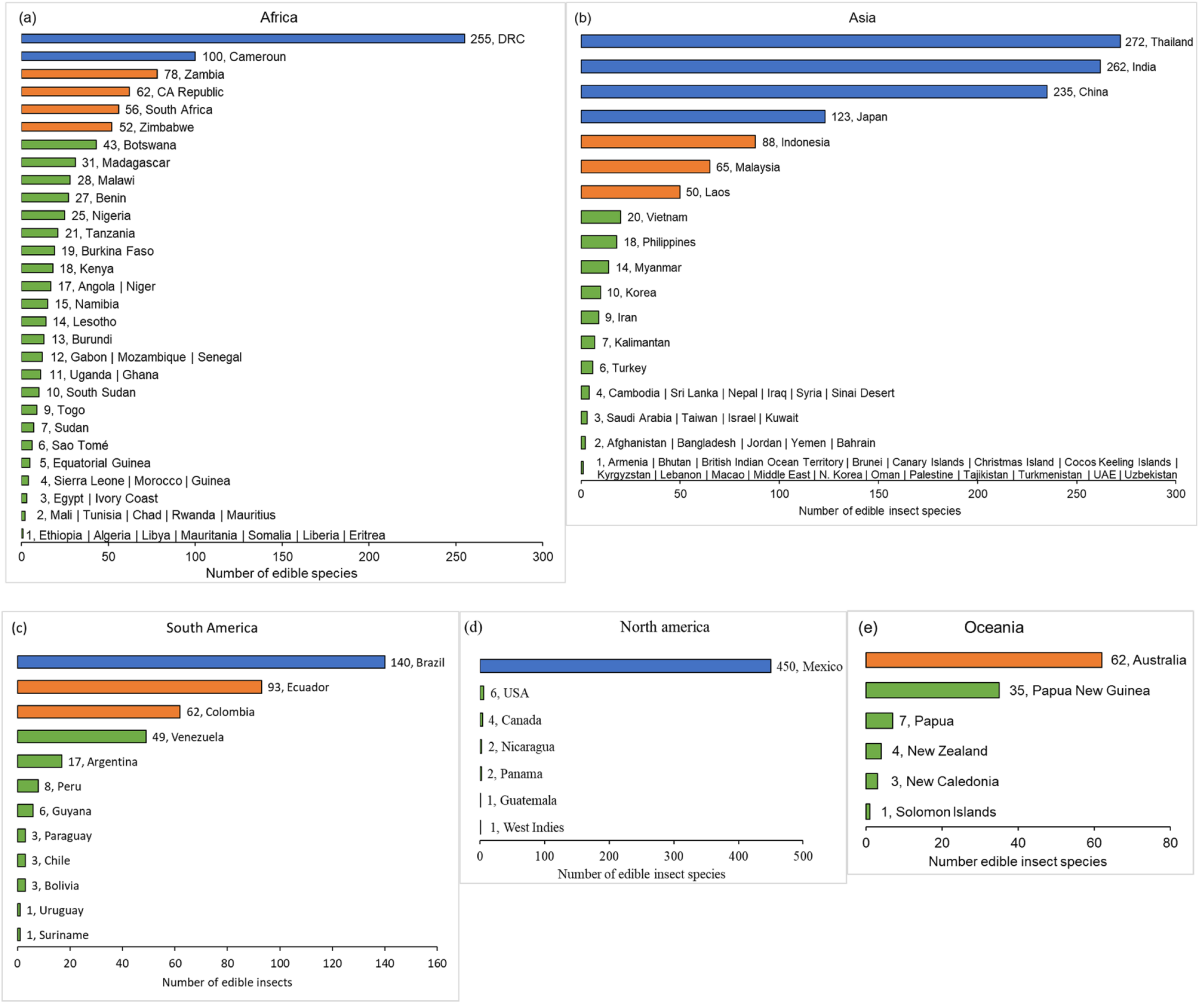
Diversity of insect species consumed in each country, grouped according to regions. *DRC* Democratic Republic of Congo. Blue =  ≥ 100 species, orange = 50–99 species and green = 1–49 species.

The consumption of insects varies significantly by country, reflecting diverse dietary preferences (Fig. 5a–e). In Africa, insects are consumed in 48 countries (Fig. 5a). The Democratic Republic of Congo (DRC) has the highest insect diversity (255 species). Other countries with substantial insect consumption include Cameroon (100 species), Zambia (78 species), the Central African Republic (62 species), South Africa (56 species), and Zimbabwe (52 species). Countries such as Botswana, South Sudan, Uganda, Ghana, Senegal, Gabon, Mozambique, Burundi, Lesotho, Namibia, Angola, Niger, Kenya, Burkina Faso, Tanzania, Nigeria, Benin, Malawi, and Madagascar have between 10 and 50 edible insect species. Togo, Sudan. Sao Tomé, Equatorial Guinea, Sierra Leone, Morocco, Guinea, Egypt, Ivory Coast, Mali, Tunisia, Chad, Rwanda, Mauritius, Ethiopia, Algeria, Libya, Mauritania, Somalia, Liberia, and Eritrea consume fewer edible insects, ranging from 1 to 9 species.

In Asia, approximately 52 countries incorporate insects into their diets (Fig. 5b). Thailand, India, and China are the leaders in insect consumption, with 272, 262 and 235 species, respectively. Japan also has a considerably diverse edible insect species (123), followed by Indonesia (88), Malaysia (65) and Laos (50). South Korea, Myanmar, the Philippines, and Vietnam consume many insects (10–20 species). The lowest numbers of edible insects (1–9 species) are consumed in Singapore, Pakistan, Qatar, Mongolia, Maldives, North Korea, Brunei, Uzbekistan, Christmas Island, Macao, Armenia, Lebanon, United Arab Emirates, Turkmenistan, Kyrgyzstan, Bhutan, Oman, Palestine, Tajikistan, British Indian Ocean Territory, Cocos Keeling Islands, the Middle East, the Canary Islands, Afghanistan, Bangladesh, Jordan, Yemen, Bahrain, Saudi Arabia, Taiwan, Israel, Kuwait, Cambodia, Sri Lanka, Nepal, Iraq, Syria, Sinai Desert, Turkey, Kalimantan, and Iran.

In South America, about 15 countries consume insects (Fig. 5c). Brazil leads in insect diversity, with 140 species, followed by Ecuador (93 species), Colombia (62 species) and Venezuela (49 species). Argentina consumes around 18 insect species, while fewer species are consumed in Peru, Guyana, Bolivia, Chile, Paraguay, Panama, Nicaragua, Suriname, Uruguay, and Guatemala.

Mexico has the highest number of edible insect species (450) in North America (Fig. 5d). In contrast, the United States of America (USA) and Canada consume six and four insect species, respectively, while Nicaragua and Panama each have two edible insect species. Only one edible insect species is consumed in Guatemala and the West Indies.

In the Oceania region, Australia leads in insect consumption, with 62 species, followed by Papua New Guinea (35 species) (Fig. 5e). Papua, New Caledonia, New Zealand, and the Solomon Islands have lower documented numbers of edible insect species, ranging from 1 to 7 species. These variations in edible insect consumption across continents and countries underscore the rich diversity of dietary practices and the importance of considering local preferences in insect-related policies and initiatives.

### Numbers of edible insect species that are common and specific to countries and continents

**Figure 6:**
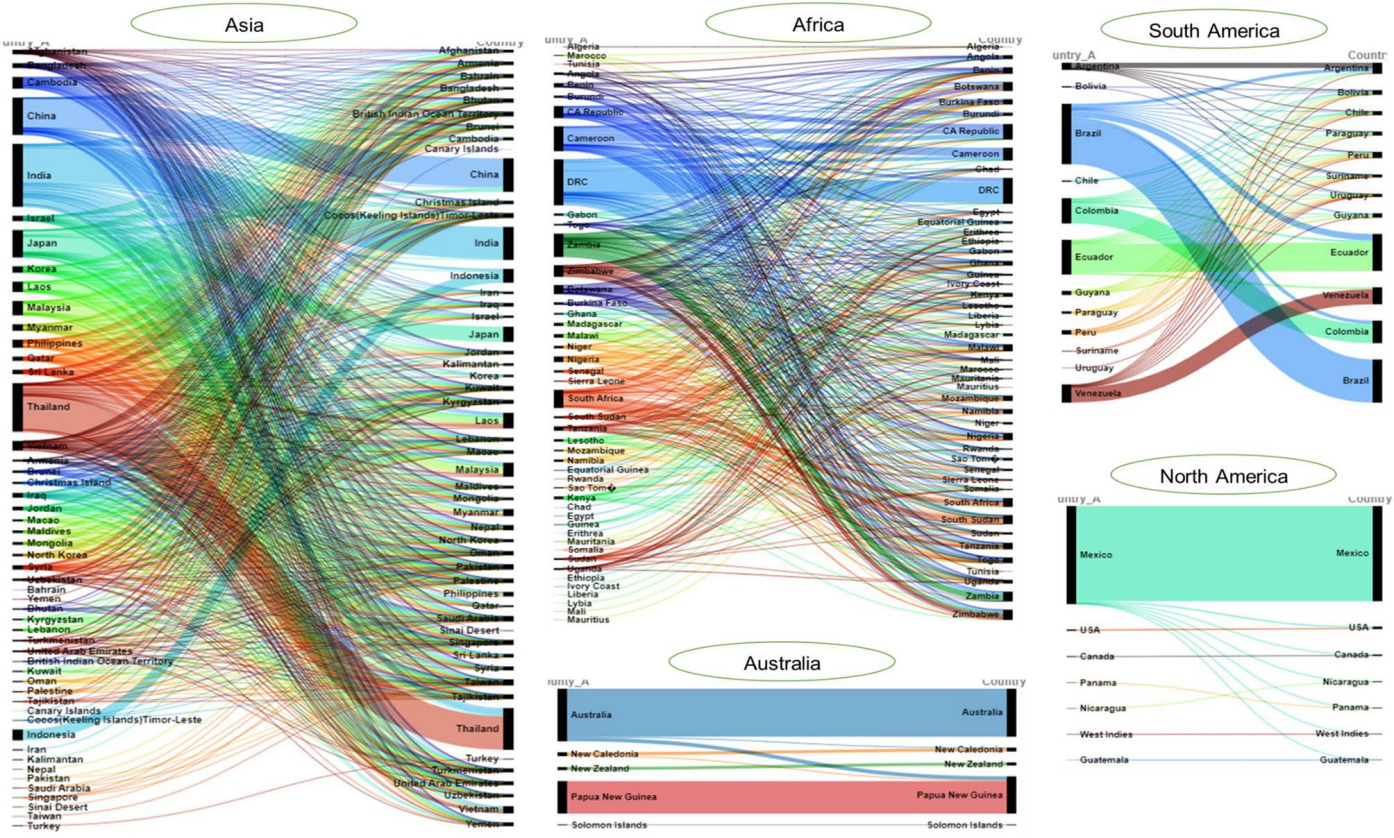
Web of transnational edible insects that are conserved and shared among countries in a continent. The thickness of a line equals the number of common insects consumed within or between countries.

The proportions of edible insect species shared among countries within each continent are illustrated in Fig. 6. Notably, many insect species are shared among countries in Asia, Africa, and South America, reflecting common dietary practices and regional preferences.

In Asia, countries such as Thailand, India and China have the highest number of edible insects, which they share with other countries. Some of the most widely shared insect species found in at least five Asian countries include *Sitophilus oryzae* L. (Coleoptera: Curculionidae)*, Schistocerca gregaria* Forsskål (Orthoptera: Acrididae), *Cybister* spp. (Coleoptera: Dytiscidae), *Hydrophilus hastatus* L. (Coleoptera: Hydrophilidae)*, Rhynchophorus ferrugineus* Olivier (Coleoptera: Curculionidae), *Hydrophilus palpalis* Brullé (Coleoptera: Hydrophilidae), *Gryllotalpa africana* Palisot de Beauvois (Orthoptera: Gryllotalpidae), *Cybister tripunctatus* Olivier (Coleoptera: Dytiscidae), *Eretes sticticus* L. (Coleoptera: Dytiscidae), *Hydrophilus cavisternum* Bedel (Coleoptera: Hydrophilidae), *Lethocerus indicus* Lepeletier & Serville (Heteroptera: Belostomatidae), *Apis dorsata* F. (Hymenoptera: Apidae)*, Apis mellifera* L. (Hymenoptera: Apidae), *Xylocopa* sp. (Hymenoptera: Apidae), *Bombyx mori* L. (Lepidoptera: Bombycidae) and *Tarbinskiellus portentosus* L. (Orthoptera: Gryllidae).

In South America, Brazil has the highest number of edible insects and shares certain species with countries such as Ecuador, Argentina, Bolivia, Chile, Peru, Surinam, Uruguay, Guyana, and Colombia. Ecuador, the second-ranking country, shares common species with Argentina, Bolivia, Chile, Paraguay, and Peru. Countries such as Guyana, Paraguay, Peru, Suriname, and Uruguay share many commonalities, while having specific edible insects unique to their regions. Some of the frequently shared insects in at least three countries in South America include *Rhynchophorus palmarum* L. (Coleoptera: Curculionidae), *Atta cephalotes* L. (Hymenoptera: Formicidae), *Macrodontia cervicornis* L. (Coleoptera: Cerambycidae), *Umbonia spinosa* F. (Homoptera: Cicadellidae), *Trigona clavipes* F. (Hymenoptera: Apidae), *Cephalotrigona capitata* Smith (Hymenoptera: Apidae), *Tetragonisca angustula* Latreille (Hymenoptera: Apidae), and *Atta sexdens* L. (Hymenoptera: Formicidae).

In Africa, the DRC, Cameroon, and Zambia emerged as being the primary leaders in edible insect consumption. The following insects are commonly shared in at least five African countries: *Schistocerca gregaria* Forsskål (Orthoptera: Acrididae)*, Acanthacris ruficornis* F. (Orthoptera: Acrididae)*, Apis mellifera* L. (Hymenoptera: Apidae)*, Macrotermes* spp*.* (Isoptera: Termitidae)*, Cirina forda* Westwood (Lepidoptera: Saturniidae), *Brachytrupes membranaceus* Drury (Orthoptera: Gryllidae), *Bunaea alcinoë* Stoll (Lepidoptera: Saturniidae), *Eumeta cervine* Druce (Lepidoptera: Psychidae)*, Nomadacris septemfasciata* Audinet-Serville (Orthoptera: Acrididae)*, Rhynchophorus phoenicis* F. (Coleoptera: Curculionidae)*, Carebara vidua* Smith (Hymenoptera: Formicidae)*, Carebara lignata* Westwood (Hymenoptera: Formicidae)*, Anaphe panda* Boisduval (Lepidoptera: Notodontidae)*, Cirina butyrospermi* Vuillet (Lepidoptera: Saturniidae)*, Dactyloceras lucina* Drury (Lepidoptera: Brahmaeidae.)*, Imbrasia ertli* Rebel (Lepidoptera: Saturniidae)*, Platysphinx stigmatica* Mabille (Lepidoptera: Sphingidae), *Zonocerus variegatus* L. (Orthoptera: Pyrgomorphidae)*, Clania moddermanni* Heylaerts (Lepidoptera: Psychidae)*, Imbrasia epimethea* Drury (Lepidoptera: Saturniidae)*, Urota sinope* Westwood (Lepidoptera: Saturniidae)*, Phymateus viridipes brunneri* Bolivar (Orthoptera: Pyrgomorphidae), *Ruspolia differens* Serville (Orthoptera: Tettigoniidae), *Macrotermes (Bellicositermes)* spp*.* (Blattodea: Termitidae)*, Epanaphe carteri* Walsingham (Lepidoptera: Notodontidae)*, Imbrasia belina* Westwood (Lepidoptera: Saturniidae)*, Striphnopteryx edulis* Boisduval (Lepidoptera: Eupterotidae), *Locusta migratoria migratorioides* Fairmaire & Reiche (Orthoptera: Acrididae), *Locustana pardalina* Walker (Orthoptera: Acrididae)*, Oryctes boas* Oryctes *boas* Fabricius (Coleoptera: Scarabaeidae)*, Hypotrigona gribodoi* Magretti (Hymenoptera: Apidae), *Meliponula bocandei* Spinola (Hymenoptera: Apidae)*, Bunaea caffraria* Hübner (Lepidoptera: Saturniidae), *Gynanisa maja* Klug (Lepidoptera: Saturniidae), *Heniocha dyops* Maassen (Lepidoptera: Saturniidae), *Heniocha marnois* Rogenhofer (Lepidoptera: Saturniidae)*, Lophostethus demolinii* Angas (Lepidoptera: Sphingidae)*, Acanthacris ruficornis citrina* Serville (Orthoptera: Acrididae), *Anacridium burri* Dirsh (Orthoptera: Acrididae)*,* and *Goliathus* spp. (Coleoptera: Scarabaeidae). Generally, many edible insects found in the DRC are common in Central, Southern, Eastern and West African countries. Cameroon and Zambia share edible insects with Central African countries and neighbouring nations. Additionally, South Africa exhibits several commonalities with countries in the southern African region such as Zimbabwe and Malawi.

In North America and Oceania, relatively few edible insect species are shared among countries. In the Oceania region, commonly shared species between at least two countries are *Mallodon costatus* Montrouzier (Coleoptera: Cerambycidae), *Rhynchophorus bilineatus* Montrouzier (Coleoptera: Curculionidae), *Oecophylla smaragdina* F. (Hymenoptera: Formicidae)*,* and *Teleogryllus commodus* Walker (Orthoptera: Gryllidae). For a comprehensive list of commonly shared edible insects among countries and continents, please refer to Supplementary Table 1.

### Worldwide distribution of edible insects: presence does not mean consumption

**Figure 7:**
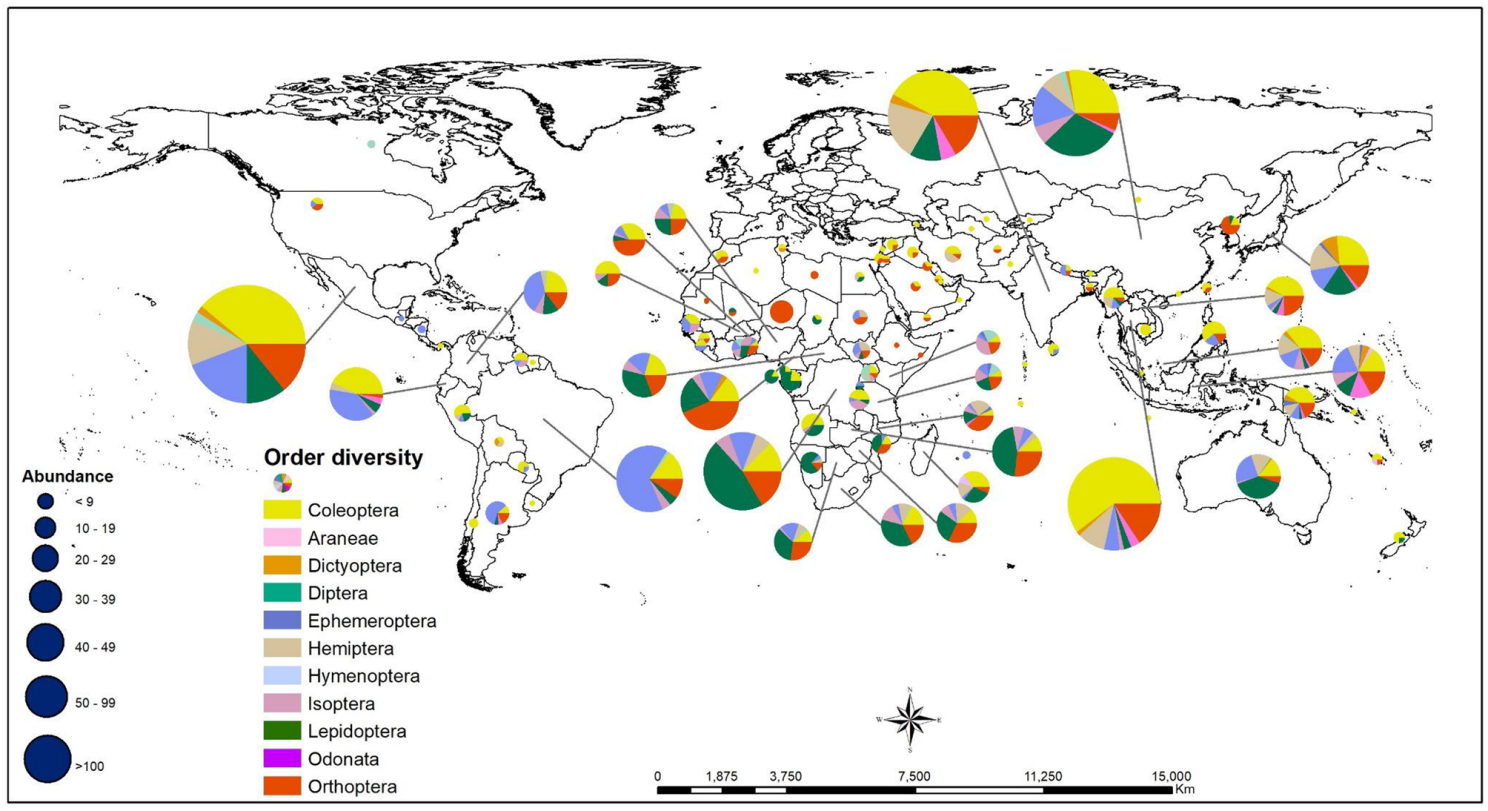
Global distribution of ‘confirmed’ edible insect species. Confirmed edible insect species imply species that have been reported in the literature. The figure was generated using QGIS geographic information system software version 3.34.3 (http://qgis.osgeo.org/).

**Figure 8:**
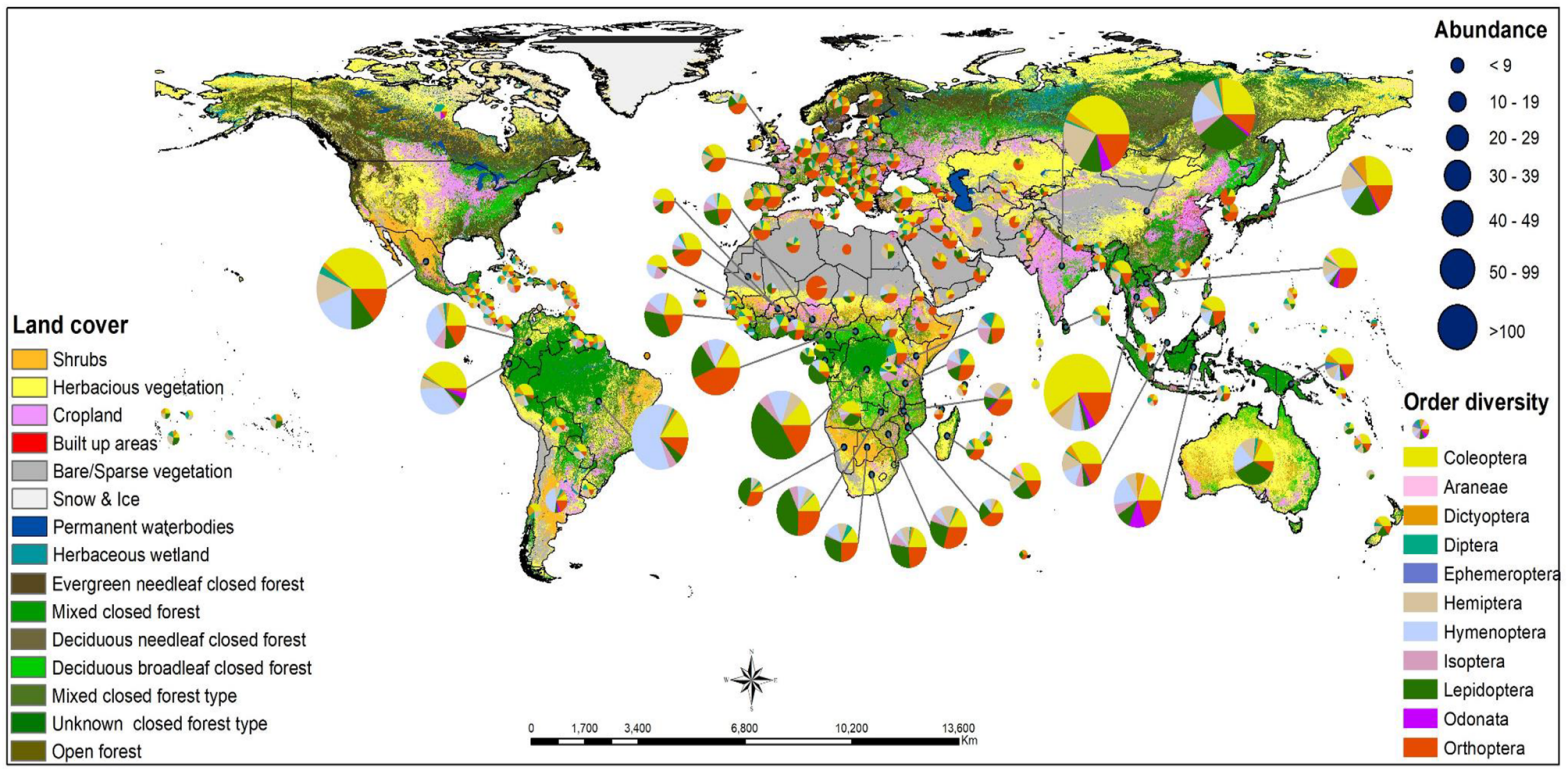
Global distribution of potentially edible insect species across different land cover classifications. Potentially edible insect species imply both confirmed and unconfirmed edible insects. The figure was generated using QGIS geographic information system software version 3.34.3 (http://qgis.osgeo.org/).

The global distribution of potentially edible insect species is illustrated in Fig. 7, showing their presence in various regions, worldwide. However, it is essential to note that the mere presence of these species does not necessarily equate to their consumption, as depicted in Fig. 8. While potentially edible insect species are found in nearly every corner of the globe, the numbers of insect species that have been confirmed as being edible are relatively limited.

The highest concentration of potentially edible insect species is primarily observed in tropical regions, with Mexico leading in number, followed by China, the DRC, India, Thailand, and Brazil. Remarkably, even those countries without a cultural tradition of insect consumption host at least one to ten potentially edible insect species. In terms of ecological distribution, areas characterised by shrubs, herbaceous vegetation and mixed closed forests tend to host the most abundant and diverse assortment of edible insects, as indicated in Fig. 8. This insight highlights the significance of considering the ecological context when evaluating the potential for edible insect utilisation in various regions worldwide.

### Proportion of consumed insect species per country

**Figure 9:**
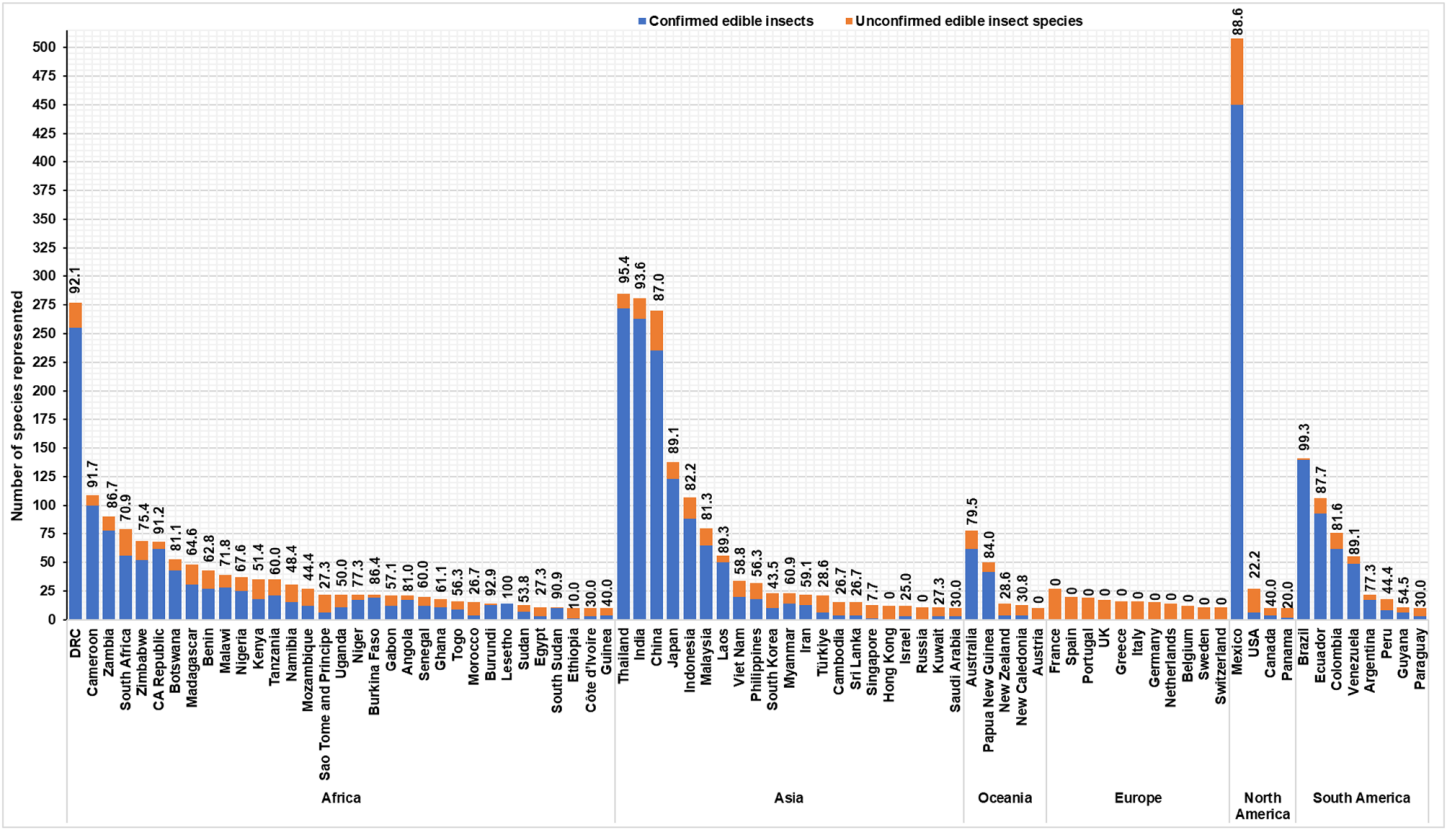
Number (diversity) of potentially edible insect species consumed elsewhere (orange) and confirmed (blue) edible insect species in each country. Only countries with more than 10 potentially edible insect species are represented here. For the values provided above, the bars represent the percentages (%) of edible insect species, based on published reports.

Figure 9 represents the diversity and proportion of “confirmed” (reported in the literature) and “unconfirmed (no literature reports) edible insects in countries where at least ten (10) species are reported. Countries with the highest proportion of consumed insect species (> 70%) are in Africa, including Lesotho, Burundi, Cameroon, the DRC, the Central African Republic, South Sudan, Zambia, Burkina Faso, Angola, Botswana, Niger, Zimbabwe, Malawi and South Africa, followed by Asia (India, China, Indonesia, Kalimantan, Sinai Desert, Thailand, Japan, Laos, Taiwan), South America (Brazil, Venezuela, Ecuador, Colombia, Argentina), and the Oceania region (Papua New Guinea, Malaysia and Australia). In North America, Mexico has the highest proportion of reported consumed insect species at 89%. The diversity of all potentially edible (unreported and reported) and the proportion of edible insects for each country is shown in Supplementary Table 2.

### Determinants of insect consumption

In order to determine the relationships between the diversity and the use and consumption of edible insects, globally, the following variables were assessed.

**Figure 10:**
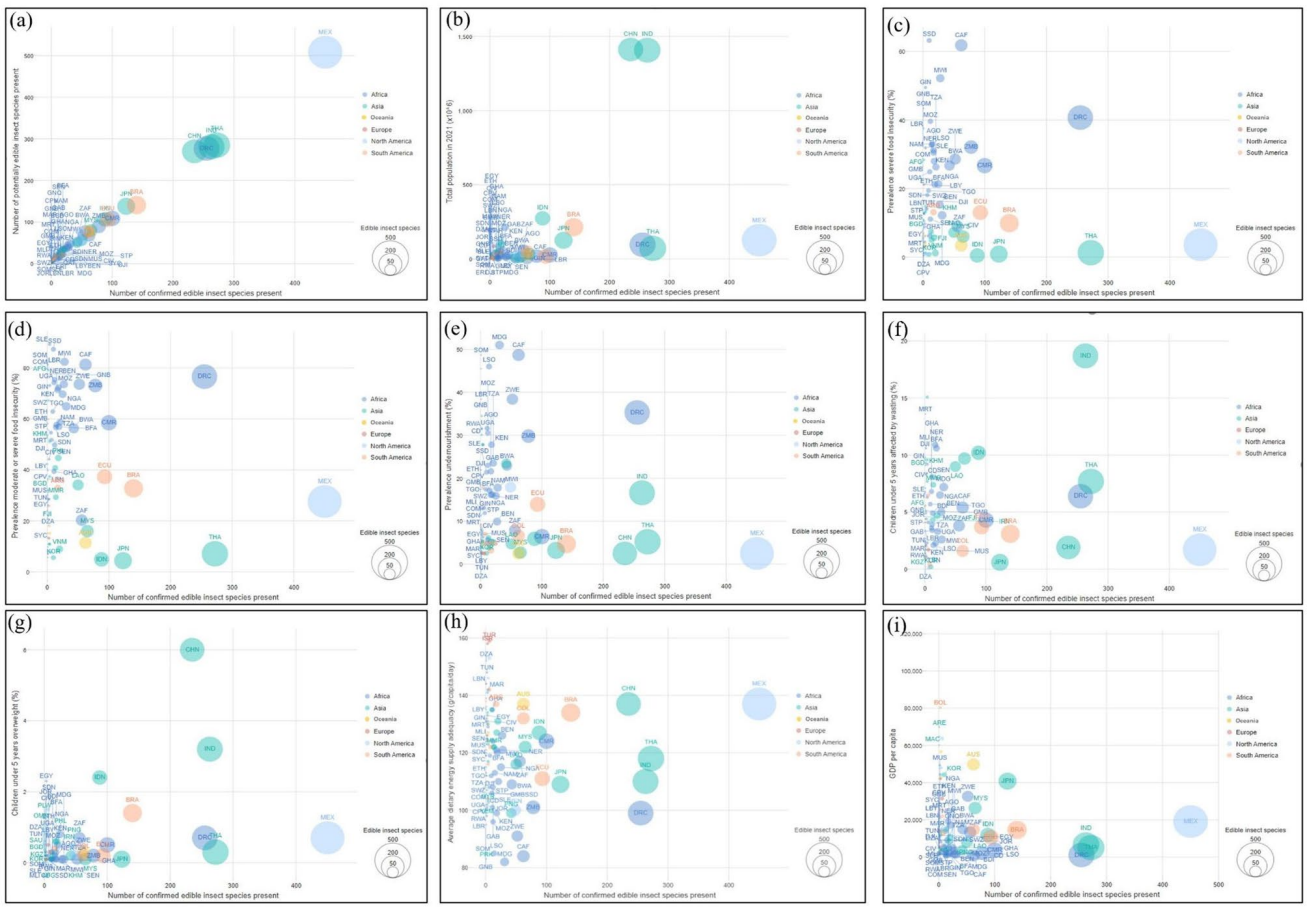
The relationships between the diversity of confirmed edible insects with the diversity of all potential edible insects (**a**), population size (**b**), food insecurity/security status (**c–g**), dietary energy supply (**h**) and GDP per capita (**i**). *AFG* Afghanistan, *ALB* Albania, *DZA* Algeria, *ASM* American Samoa, *AND* Andorra, *AGO* Angola, *ATG* Antigua and Barbuda, *ARG* Argentina, *ARM* Armenia, *ABW* Aruba, *AUS* Australia, *AUT* Austria, *AZE* Azerbaijan, *BHS* Bahamas, *BHR* Bahrain, *BGD* Bangladesh, *BRB* Barbados, *BLR* Belarus, *BEL* Belgium, *BLZ* Belize, *BEN* Benin, *BMU* Bermuda, *BTN* Bhutan, *BOL* Bolivia, *BIH* Bosnia and Herzegovina, *BWA* Botswana, *BRA* Brazil, *VGB* British Virgin Islands, *BRN* Brunei, *BGR* Bulgaria, *BFA* Burkina Faso, *BDI* Burundi, *KHM* Cambodia, *CMR* Cameroon, *CAN* Canada, *CPV* Cape Verde, *CYM* Cayman Islands, *CAF* Central African Republic, *TCD* Chad, *CHL* Chile, *CHN* China, *COL* Colombia, *COM* Comoros, *CRI* Costa Rica, *CIV* Cot’ d’Ivoire, *HRV* Croatia, *CUB* Cuba, *CYP* Cyprus, *CZE* Czechia, *DNK* Denmark, *DJI* Djibouti, *DOM* Dominican Republic, *DRC* Democratic Republic of Congo, ECU Ecuador, *EGY* Egypt, *SLV* El Salvador, *GNQ* Equatorial Guinea, *ERI* Eritrea, *EST* Estonia, *SWZ* Eswatini, ETH Ethiopia, *FJI* Fiji, *FIN* Finland, *FRA* France, *GAB* Gabon, *GMB* Gambia, *GEO* Georgia, *DEU* Germany, *GHA* Ghana, *GIB* Gibraltar, *GRC* Greece, *GRD* Grenada, *GUM* Guam, *GTM* Guatemala, *GIN* Guinea, *GNB* Guinea-Bissau, *GUY* Guyana, *HND* Honduras, *HKG* Hong Kong, *HUN* Hungary, *IND* India, *IDN* Indonesia, *IRN* Iran, *IRL* Ireland, *IMN* Isle of Man, *ISR* Israel, *ITA* Italy, *JAM* Jamaica, *JPN* Japan, *JOR* Jordan, *KAZ* Kazakhstan, *KEN* Kenya, *KIR* Kiribati, *XKX* Kosovo, *KWT* Kuwait, *KGZ* Kyrgyz Republic, *LAO* Lao PDR, *LVA* Latvia, *LBN* Lebanon, *LSO* Lesotho, *LBR* Liberia, *LBY* Libya, *LIE* Liechtenstein, *LTU* Lithuania, *LUX* Luxembourg, *MAC* Macao SAR, China, *MDG* Madagascar, *MWI* Malawi, *MYS* Malaysia, *MDV* Maldives, *MLI* Mali, *MLT* Malta, *MHL* Marshall Islands, *MRT* Mauritania, *MUS* Mauritius, *MEX* Mexico, *FSM* Micronesia, Fed. States, *MDA* Moldova, *MNG* Mongolia, *MNE* Montenegro, *MAR* Morocco, *MOZ* Mozambique, *MMR* Myanmar, *NAM* Namibia, *NPL* Nepal, *NLD* Netherlands, *NCL* New Caledonia, *NZL* New Zealand, *NIC* Nicaragua, *NER* Niger, *NGA* Nigeria, *PRK* North Korea, *MNP* Northern Mariana Islands, *NOR* Norway, *OMN* Oman, *PAK* Pakistan, *PLW* Palau, *PAN* Panama, *PNG* Papua New Guinea, *PRY* Paraguay, *PER* Peru, *PHL* Philippines, *POL* Poland, *PRT* Portugal, *PRI* Puerto Rico, *QAT* Qatar, *ROU* Romania, *RUS* Russian Federation, *RWA* Rwanda, *STP* Sao Tome and Principe, *SAU* Saudi Arabia, *SEN* Senegal, *SRB* Serbia, *SYC* Seychelles, *SLE* Sierra Leone, *SGP* Singapore, *SVK* Slovak Republic, *SVN* Slovenia, *SLB* Solomon Islands, *SOM* Somalia, *ZAF* South Africa, *KOR* South Korea, *SSD* South Sudan, *ESP* Spain, *LKA* Sri Lanka, *SDN* Sudan, *SUR* Suriname, *SWE* Sweden, *CHE* Switzerland, *SYR* Syrian Arab Republic, *TJK* Tajikistan, *TZA* Tanzania, *THA* Thailand, *TLS* Timor-Leste, *TGO* Togo, *TON* Tonga, *TTO* Trinidad and Tobago, *TUN* Tunisia, *TUR* Türkiye, *TKM* Turkmenistan, *TCA* Turks and Caicos Islands, UGA Uganda, *UKR* Ukraine, *ARE* United Arab Emirates, *GBR* United Kingdom, *USA* United States, *URY* Uruguay, *UZB* Uzbekistan, *VEN* Venezuela (Republic of Bolivarian), *VNM* Vietnam, *YEM* Yemen, *ZMB* Zambia, *ZWE* Zimbabwe.

- Number of potentially edible insects—The abundance of confirmed edible insect species had a positive and significant correlation with the number of potentially edible insect species present in a country (R = 1, p < 0.001, Fig. 10a). Mexico, Thailand, India, China, and the DRC show the highest likelihood of edible insect consumption.
- The number of edible insects directly correlates with countries’ population sizes (R = 0.53, p < 0.001, Fig. 10b). India, China, Indonesia, and Brazil are among the countries with high numbers of edible insects and large populations. Interestingly, countries such as Mexico, Thailand, and the DRC, with population sizes of between 70 and 130 million, also had high numbers of insects being consumed.
- Prevalence of severe food insecurity—there was no significant correlation between the abundance of confirmed edible insect species and food insecurity/security parameters, such as the prevalence of moderate to severe food insecurity (R = 0.067, p = 0.44) and the prevalence of severe food insecurity (R = 0.086, p = 0.32, Fig. 10c,d). South Sudan, Central Africa, Malawi, Guinea, Guinea Bissau, Somalia, Tanzania, and Mozambique are some countries with serious food insecurity issues, but relatively low numbers of edible insects. The DRC has a relatively high prevalence of food insecurity issues, but high numbers of edible insects. On the other hand, Mexico, Thailand, Japan, and India have a low prevalence of food insecurity issues, with a high number of edible insects.
- Prevalence of undernourishment—The prevalence of undernourishment correlated positively with edible insect presence, but was not significant (R = 0.053, p = 0.5, Fig. 10e). Madagascar, Central African Republic, Somalia, and Lesotho were some countries with the highest prevalence of undernourishment, but with fewer than 100 edible insects. The DRC and India each have a relatively high prevalence of undernourishment, but with more than 100 edible insects. Meanwhile, China, Thailand and Mexico have a low prevalence of undernourishment, with many edible insects.
- Percentage of children under five years affected by wasting—Child wasting, which refers to a thin body suffered as a result of recent rapid weight loss or failure to gain weight, did not correlate with the number of edible insects consumed (R = 0.14, p = 0.17, Fig. 10f).
- Number of edible insects and children under five years overweight—There was a positive significant correlation between the abundance of confirmed edible insect species and children under 5 years who are overweight (R = 0.47, p < 0.001, Fig. 10g).
- Dietary supply adequacy—Regarding dietary supply adequacy, which is a measure of food security that considers the average supply of calories for food consumption, the number of edible insect species had a negative but insignificant correlation with the average dietary energy supply adequacy (R =  − 0.088, p = 0.26, Fig. 10h). Countries with the highest dietary supply adequacy, e.g., Turkey, Israel, Tunisia, and Lebanon, do not consume or, at most, consume few numbers of edible insects. In contrast, countries with the highest number of edible insects do not necessarily have satisfactory dietary energy supply adequacy, e.g., Mexico, Thailand, India, DRC, and China.
- GDP per capita—The number of edible insect species had a negative but insignificant correlation with GDP per capita (R =  − 0.12, p = 0.11, Fig. 10i). Generally, countries with high GDP tend to have little culture of eating insects. Countries with fewest documented edible insects (1–10 species), e.g., Bolivia, United Arab Emirates, Macao SAR, United States, Brunei, New Caledonia, Bahrain, Canada, South Korea and New Zealand have the highest GDP per capita, whereas countries such as India, Thailand, Nigeria, Kenya, Cameroon, Papua New Guinea, Benin, Zambia, Malawi, Madagascar, and DRC with the highest number of edible insects (> 30 species) have low (< 10,000) GDP per capita. However, a few countries, e.g., Japan, China, and Malayasia consume many edible insects and have a relatively high GDP per capita.

The multivariate regression indicated that number of confirmed, and GDP per capita were the only variables that significantly predicted practice of insect consumption (Table 1).

**Table 1:** Regression coefficients for predicting insect consumption practices (number of confirmed edible insects).

Variable	Estimated coefficient (β)	Standard error (β)	t-value	p-value
Intercept	− 2.0807	16.1721	− 0.129	0.898
Number of potentially edible insect species	0.9270	0.0104	89.38	< 0.001
Total population	0.0200	0.0282	0.709	0.484
GDP per capita	− 0.0003	0.0001	− 2.389	0.023
Children under 5 years overweight (%)	− 4.0013	4.3097	− 0.928	0.360
Children under 5 years affected by wasting (%)	− 0.0064	0.3502	− 0.018	0.986
Prevalence moderate or severe food insecurity (%)	− 0.0911	0.1090	− 0.836	0.410
Prevalence severe food insecurity (%)	0.1153	0.1532	0.753	0.457
Prevalence undernourishment (%)	− 0.1105	0.1972	− 0.560	0.579
Average dietary energy supply (g/capita/day)	0.0150	0.1216	0.123	0.903
Adjusted R^2^ = 0.9957				

## Discussion

Despite constituting a small fraction of the class, edible insects are a key component of the global food system and could significantly address nutritional insecurities. Our extensive inventory reveals the consumption of over 2205 species from 24 distinct orders of insect and one order of arachnids, across 128 countries, surpassing earlier records of 1900 species in 113 countries^12,31^. This suggests the likelihood of the existence even more insect orders among insect species being consumed globally, which await documentation. Understanding this distribution of edible insects across orders provides valuable insights into their consumption patterns and ecological significance, offering a foundation for further research and policy considerations in sustainable food systems. The dominant insect orders among edible species worldwide are Coleoptera, Hymenoptera, Lepidoptera and Orthoptera. This distribution aligns with the fact that Asia, home to over 932 edible insect species, primarily relies on Coleoptera as the key edible insect group. Coleoptera’s prominence could also be attributed to its status as being the most prominent order among insects, known for its crude protein content^12,43^, and ease of collection and preparation. Similar findings have been reported by van Huis^12^, where beetles (Coleoptera) accounted for the most-consumed insect group, followed by caterpillars (Lepidoptera), bees, wasps, and ants (Hymenoptera), and grasshoppers, locusts, and crickets (Orthoptera). Lepidopterans are consumed the most in Africa, while Hymenopterans are consumed the most in South America. Coleoptera, Hymenoptera, Lepidoptera and Orthoptera are the most consumed across the continents because of the geographical presence of potentially edible insects (both reported and unreported) under these orders. Kelemu et al.^30^ reported similar results for Lepidopteran in Africa. Lepidoptera constitute one of the most important orders in terms of abundance and diversity, with more than 188,359 species being described in more than 4000 genera (https://www.gbif.org/fr/species/797/metrics). The most-consumed stages are the caterpillars, which depend on plants for their growth and survival. Edible caterpillars contribute to food security and income in many rural areas where they are harvested and processed for the market, and they contribute to economic growth^44–48^. Some species are also consumed at the pupal stage^47,49^.

We found that the orders with the least-consumed numbers of insect species were Hemiptera, Isoptera, Odonata, Diptera, Dictyoptera, Ephemeroptera, Plecoptera, Trichoptera, Phasmida, Megaloptera, Dermaptera and Psocoptera. The lowest numbers of edible insect species under these orders have been previously reported^12^. We speculate that members of these insect orders are poisonous because of the toxins they produce; therefore, relatively low numbers are deemed edible. The estimation of the dominance of edible insects by biomass has scarcely been considered. For instance, although termite species consumed in Africa are relatively few, they are probably among the most widely eaten insects on the continent^50^.

Our results show that edible insects are widely distributed and found in almost all climatic conditions, while vegetation supports the existence of edible insect species in most parts of the world. According to van Huis^12^, insect consumption is highly recognised in the tropics for the following reasons. (1) Insects tend to be diverse in the tropics, which facilitates harvesting; (2) insects, such as desert locusts, winged termites, caterpillars and crickets, often congregate in significant numbers in the tropics and can be collected during a single harvest; (3) unlike in temperate zones, a variety of edible insect species can be found year-round in the tropics; (4) the harvesting places and times for many insect species are predictable; and (5) the tropics favour many hosts, such as palms (for palm weevils) and bamboo (for bamboo caterpillars), or favour the existence of the edible insects themselves (for example soldier termites in termite mounds). However, the most intriguing question is as to how people in the tropics came to select specific insects, from among the vast variety of insect species available. Cultural and religious practices heavily influence insect consumption, and insects are commonly consumed as a food source in many regions of the world^12^. Culture, influenced by the environment, history, community structure, human endeavour, mobility, and politico-economic systems, defines the rules on what is edible and what is not. The acceptance or rejection of entomophagy is a question of culture^12,51–53^. In many countries, people still view eating insects as a disgusting and primitive behaviour. This attitude has resulted in the neglect of insects in agricultural research^12,51,54^. Nonetheless, recent advances in edible research for development are expanding consumption, and the trend is likely to continue.

In many parts of the world, eating insects is a cultural heritage that has been transmitted from generation to generation, from before the advent of modern civilisation. Many tropical and sub-tropic communities have long traditions of entomophagy^13^. Insects are considered as part of their normal diets for over 3000 ethnic groups, mainly in African, Asian, and Latin American countries^12^. For instance, Niassy^51^ showed that about 30 ethnic groups in 12 sub-Saharan countries have a simple language resemblance for naming edible termites, that is, “Tsiswa”, “Chiswa”, “Chintuga”, “Inswa”, “Iswa”, “Sisi”, “Ishwa” or “Esunsun”.

Our findings show that insects are consumed in nearly all African countries. Among the regions, we found the highest diversity of consumed insects in the Central African region (the DRC, Cameroon, and the Central African Republic), followed by Southern Africa (Zambia, South Africa, Zimbabwe, and Botswana). This corroborates the finding of Kelemu et al.^30^, where the authors reported that the Central African region had the highest number of confirmed edible insects, followed by the Southern African region. A more recent study by Egonyu et al.^55^ documented the findings that the DRC, Cameroon, South Africa, Zambia, and Zimbabwe are countries in Africa with prominent histories of entomophagy.

In Asia, we found that the most considerable diversities of insects (more than 50 species) are consumed in Thailand, India, and China, followed by Japan, Indonesia, Malaysia, and Laos. The top five countries with the highest diversities of edible insect species in South America were Brazil, Ecuador, Colombia, Venezuela, and Argentina. In the Oceania region, the highest diversity of edible insects was observed in Australia and Papua New Guinea. In North America, Mexico is the most prominent for insect consumption (over 450 species), while the USA, Canada, Nicaragua, Panama, Guatemala, and West Indies have only between 1 and 6 edible insect species. According to Van Huis et al.^12^, the reasons why insects are not consumed mainly in Western countries include: (1) eating insects is considered disgusting; (2) high neophobia; (3) lack of information regarding edible insect products; (4) lack of prior experience of eating insects; and (5) general disinterest and indifference. As a result of the industrial revolution in Europe and increased urbanisation, most people moved to urban areas to find work^56^, thereby distancing themselves from nature. However, the negative perception of entomophagy is gradually changing in Western countries, where insects have not been traditionally recognised as food^57,58^. For instance, the consumption of insects in Europe began recently, in the twentieth century, although it is not yet widespread^59^. Between 2021 and 2023, the European Food Safety Authority (EFSA) approved the selling and consumption of migratory locust *Locusta migratoria* L. (Orthoptera: Acrididae), house cricket *Acheta domesticus* L. (Orthoptera: Gryllidae), yellow mealworm *Tenebrio molitor* L. (Coleoptera: Tenebrionidae), and lesser mealworm *Alphitobius diaperinus* Panzer (Coleoptera: Tenebrionidae) as “novel food” for humans in European nations (https://ipiff.org/insects-novel-food-eu-legislation-2/).

Generally, the majority of insect species are consumed in the African tropics, the American tropics (comprising only Mexico in North America, Central America, the Caribbean islands and the top half of South America, including Colombia, Ecuador, Peru, Bolivia, Colombia, Venezuela, Guyana and Suriname, and the northern portions of Chile, Argentina, Paraguay and Brazil) and the Asian tropics (Bangladesh, Cambodia, India, Indonesia, Malaysia, the Philippines, Sri Lanka, Thailand and Vietnam). We also observed some countries with many edible insect species in subtropical Asia (China), subtropical African countries (South Africa and Namibia), and subtropical Oceania countries. We found these areas to be characterised by a heavy presence of shrubs, herbaceous vegetation, and mixed closed forests, which can harbour diverse edible insect species.

Despite their presence in almost all countries, we observed that the numbers of insect species confirmed to be edible were comparatively low, with some countries having no record of insect consumption. For instance, 21 potential edible insect species are found in Türkiye, with only six species being reported as being edible. In Italy and Germany, about 16 and 15 potentially edible species are present, respectively, with none being reported as edible.

Our results showed overlaps in the species of edible insects between/among countries, especially in Asia, Africa, and South America. The top 11 insects with global overlaps include *Schistocerca gregaria* Forskål (Orthoptera: Acrididae)*, Gryllus bimaculatus* De Geer (Orthoptera: Gryllidae)*, Macrodontia cervicornis* L. (Coleoptera: Cerambycidae), *Stenodontes damicornis* L. (Coleoptera: Cerambycidae), *Cybister* spp. (Coleoptera: Dytiscidae), *Oryctes rhinoceros* L. (Coleoptera: Scarabaeidae)*, Rhynchophorus ferrugineus* Oliv. (Coleoptera: Curculionidae), *Rhynchophorus palmarum* L. (Coleoptera: Curculionidae)*, Nezara viridula* L. (Hemiptera: Pentatomidae)*, Macrotermes (Bellicositermes)* sp. (Isoptera: Termitidae), *Nomadacris septemfasciata* Serville (Orthoptera: Acrididae)*,* and *Gryllotalpa africana* F. (Orthoptera: Gryllotalpidae). These findings highlight the global and regional overlaps and variations in edible insect consumption and the importance of considering these shared species in discussions surrounding dietary diversity and cultural practices. Overlaps among countries and continents of edible insect species can be attributed to potential similarities in history, ethnic communities, land cover properties, and climatic conditions that favour the existence of certain species of edible insects. Niassy^51^ established the similarity in vernacular names between edible insects in East and Southern African countries. We found that countries with high population sizes tend to consume insects. This is observed in India, China, Indonesia, Thailand, Japan, Brazil, South Africa, Papua New Guinea, Malaysia, and Australia.

Edible insects can be a key entry topic and major resource for addressing food and nutrition security. For instance, countries with acute food insecurities, such as South Sudan, the Central African Republic and Malawi, rely largely on seasonal harvesting of edible insects. This could be linked to the political crises and prolonged dry spells in those countries. However, insect consumption was not found to be related to undernourishment, confirming the cultural and religious dimensions. Madagascar, the Central African Republic, Somalia, and Lesotho are some of the countries identified with the highest prevalence of undernourishment but consumed fewer than 100 edible insect species. At the same time, the DRC and India have a relatively high prevalence of undernourishment, but with more than 100 edible insect species. Child wasting was not significantly correlated with insect consumption, whereas child overweight was positively correlated. Countries with higher child-wasting rates consume fewer than 50 insect species, compared with countries with lower child-wasting rates that consume more than 50 insects. In countries such as India and Thailand, child wasting is high, yet over 200 edible species are consumed, whereas Mexico has low child wasting, but with more than 400 insect species being eaten. Child wasting is also high in Mauritania, Ghana, Niger, Mali, Burkina Faso, Djibouti, Guinea, and Sudan, but fewer than 50 insect species are consumed.

On the other hand, we found a positive significant correlation between overweight children and insect consumption. China, India, and Indonesia are some countries with relatively high percentages of acute children overweight and consuming more than 200 insect species. Whereas countries with low percentage of acute children overweight (e.g., DRC, Thailand, Mexico, Japan, Ghana) consumed relatively high number of insects. However, this analysis was based on national average data from FAOSTAT. Further analysis based on child overweight information on specific state or region of the country, may provide a clearer picture.

A negative correlation between insect consumption and GDP per capita generally shows that low-income countries have largely embraced the culture of “entomophagy”; however, except for some of the countries, such as China, India, and Indonesia.

Countries with a higher average dietary energy supply adequacy, e.g., Türkiye, Israel, Tunisia, and Lebanon, consume few edible insect species. Countries with the most edible insect species have relatively good dietary energy supply adequacy, e.g., Mexico, Thailand, India, the DRC, and China. This finding is debatable and demonstrates the point that the eating of insects is unrelated to archaism and lack of civilisation. Economically advanced countries could still practise cultural heritage, such as insect consumption, while developing modern technologies for edible insect mass rearing and advanced processing. Countries with the highest revenues per capita, e.g., Bolivia, the United Arab Emirates, Macao SAR, Mauritius, and Korea, consume fewer insects, whereas countries with the lowest revenues consume more insects. The lack of consumption could be attributable to low diversity and the absence of insect-eating habits or culture. However, countries such as Japan and Australia, with a relatively high GDP per capita, consume many edible insects. Edible insects are viewed as providing a new source of protein, not necessarily for energy. However, in countries where people are suffering from underproduction of calorie-rich foods (e.g., staples and edible insects), the livelihood opportunities could be balanced with agricultural productivity. Our study shows that insects are a valuable food source for both low-income and high-income countries, across the globe. Given favourable climate conditions and vegetation, edible insects could be traded from low-income to non-consuming countries in an improved and sustainable food system and economic value chain.

## Conclusion

Edible insects have been part of the traditional diet of many people in the tropics and subtropics. We found that over 2205 insect species are consumed in 128 countries, and the orders with the most-reported edible species are Coleoptera, Hymenoptera, Lepidoptera and Orthoptera. We report that over 932, 529, 464, 300 and 107 edible insect species are found in Asia, North America, Africa, South America, and Oceania, respectively. The countries with at least 100 reported edible insect species are Mexico (450), Thailand (272), India (262), the DRC (255), China (235), Brazil (140), Japan (123), and Cameroon (100). Most edible insects have regional overlaps, while some have continental overlaps, which can be associated with climatic conditions, especially along the tropics and subtropics, land cover properties, and the histories of entomophagy among ethnic groups. Despite the presence of edible insects in some Western countries, insect consumption remains comparatively low. Edible insects have many benefits related to food and nutrition needs, livelihoods, economic development, social integration, and the environment, contributing significantly to achieving nearly all United Nations Sustainable Development Goals.

Despite our attempt to document edible insects of the world, the striking question is as to whether the current scientifically available data accurately represents all species of edible insects eaten, worldwide. Albeit with caveats, we speculate that many or some other edible insects are being eaten, but which have not been recorded in the literature. Therefore, this paper provides insights into estimates of the diversity of edible insects, their distribution, and commonalities across countries, continents, and the globe. Whereas this study sheds some light on some of the potential drivers for the occurrence, overlaps, and edibility status of different insects, there is also a need to document proportion of country’s population that is “entomophagous” and promote culture of eating insects.

## Methodology

### Data sources

We drew upon various published sources to construct a comprehensive inventory of global edible insects. These sources collectively provided a rich overview of the global edible insect diversity. Our data collation process began with an extensive review of the literature. We consulted a variety of studies and publications that have catalogued edible insects, worldwide. Notable sources included Jongema^31^, who compiled a list of approximately 2000 edible insects from across the globe, and van Huis^60^, who identified 250 edible insect species specifically in Africa. Other significant contributions have come from Kelemu et al.^30^, who provide an updated list of 460 edible insects in Africa; Ramos-Elorduy et al.^61^, who documented 549 species in Mexico; and Feng et al.^62^, who recorded 177 species in China. In our efforts to comprehensively document edible arthropods, we also considered arachnids, such as spiders, which fall within this broader category and have been reported in studies on edible insects (such as van Huis et al.^12^; Jongema^31^). This inclusive approach ensures a more comprehensive representation of edible species within this category. The global land cover dataset offers valuable insights into the Earth’s land cover types and is instrumental in assessing the environmental conditions suitable for various insect species. This information was sourced from the Copernicus Land Service library (https://land.copernicus.eu/global/products/lc). We gathered additional data for each country from reputable sources, including the Food and Agriculture Organization (FAO) (https://www.fao.org/faostat/en/#home). This supplementary data encompassed key factors such as population, income levels [measured by gross domestic product (GDP) per capita], and food and nutritional insecurity indicators. These factors are essential for contextualising the relationship between edible insects and socio-economic variables.

### Data compilation and verification

The resulting datasets, encompassing information on potentially edible insect species, along with details of the countries where they are found, were meticulously organised and recorded in a structured format through using the Microsoft Excel application. Any duplicate entries were identified and eliminated throughout this process to ensure data accuracy and consistency. We undertook a rigorous verification process to determine the presence of the listed insect species in various countries and territories. This verification involved systematic searches of reputable data repositories, such as the Global Biodiversity Information Facility (GBIF) and iNaturalist. These platforms are recognised for their extensive and reliable collections of biodiversity data.

### Data synthesis, analysis, and visualisation

The compiled datasets, now enriched with verified presence information, were processed, and synthesised into informative formats, including graphs and tables. This step aimed to make the data more accessible for applying analytics. In this context, multiple tools and applications were used. Geographic information system QGIS 3.34.3 software (http://qgis.osgeo.org/) was used to combine the distribution data of potential edible insect species with the global land cover dataset, and carry out the clustered analysis, linking the presence of edible insects with specific land cover types and environmental conditions. Prior to this analysis, we conducted a categorisation, considering certain species as being potentially edible. These insects lack documented consumption records in a particular country or territory but have been reported as edible in other countries. This distinction is important as it highlights the potential for expanding insect-based diets to regions where these species are not traditionally consumed. Through this analysis, we generated maps, which help to visualise the geographical distribution of edible insects, allowing us to identify regions where they are commonly consumed and those where they are potentially viable as future food sources.

We employed a generic approach to assess the prevalence of edible insects in different regions to help us to understand the extent to which edible insects were commonly consumed. We calculated the percentage of edible insects as the proportion of confirmed edible insect species, over the total number of potentially edible insects recorded in each country (or territory).

$$Proportion \,of\, Edible\, Insects = \frac{Number\, of \,Reported \,Edible\, Species }{Number\, of\, Repoted \,and\, Potentially\, Edible \,Species} \times 100.$$

Pearson’s correlation coefficient was used to estimate the strength and direction of relationships between variables. Further, a multivariate regression analysis was performed, using the numbers of reported edible insects as the predictors, with population size, food insecurity parameters and GDP constituted the dependent variable. Correlation and multivariate regression analysis was performed by using R Software^63^.

To visualise the transnational commonalities and specificities of insect species within continents, we employed the data visualisation tool RawGraphs (https://app.rawgraphs.io/). Sankey diagrams generated through this platform clearly represented how certain insect species are distributed across continents, offering insights into both shared and unique edible insect species within these regions. To render the relationships between these variables more accessible, we used bubble plots. These graphical representations were generated through using Displayr (https://westeurope.displayr.com), an online data visualisation tool. Bubble plots effectively display multivariate data using bubbles of varying sizes and colours to represent different variables, making it easier to identify patterns, trends, and correlations.

### Supplementary Information

Supplementary Tables.

## Data Availability

The datasets that support the findings of this study are available on request to the corresponding author.
