# Supplementary material for: The global atlas of edible insects: analysis of diversity and commonality contributing to food systems and sustainability
Source: Sci Rep. 2024 Feb 29;14:5045. doi: 10.1038/s41598-024-55603-7 (PMC10904393; doi:10.1038/s41598-024-55603-7)
Supplement: Supplementary file 1 — Supplementary Tables. [file 41598_2024_55603_MOESM1_ESM.docx]

**Supplementary materials**

**Supplementary Table 1**

Common insects consumed among continents (plus countries)

| **Order** | **Family** | **Species** | **Continent 1** | **Continent 2** | **Continent 3** | **Continent 4** |
| --- | --- | --- | --- | --- | --- | --- |
| Orthoptera | Gryllidae | *Acheta domesticus* (L.) | Asia (Thailand) | N. America (Mexico) |  |  |
| Coleoptera | Cerambycidae | *Macrodontia cervicornis* (L.) | Asia (India) | S. America (Brazil, Guyana, Paraguay, West Indies, Jamaica, Ecuador) |  |  |
| Coleoptera | Cerambycidae | *Stenodontes damicornis* (L.) | Asia (China) | South America (Brazil, Guyana, West Indies) |  |  |
| Coleoptera | Curculionidae | *Rhynchophorus* sp. | Africa (Madagascar) | S. America (Colombia) |  |  |
| Coleoptera | Dytiscidae | *Cybister flavocinctus* (Aubé) | Asia (China) | N. America (Mexico) |  |  |
| Coleoptera | Dytiscidae | *Cybister hova* (Fairm) | Africa (Madagascar) | N. America (Mexico) |  |  |
| Coleoptera | Dytiscidae | *Cybister* sp. | Asia (Vietnam, Laos, Thailand, Cambodia, Myanmar) | N. America (Mexico) | Africa (Benin) |  |
| Coleoptera | Dytiscidae | *Dytiscus habilis* (Say) | Asia (China, Japan) | N. America (Mexico) |  |  |
| Coleoptera | Dytiscidae | *Eretes sticticus* (L.) | Asia (Myanmar, India, Thailand) | Africa (Kenya) |  |  |
| Coleoptera | Scarabaeidae | *Dicranrrhina micans* (Drury) | Africa (Burundi) | S. America (Ecuador) |  |  |
| Coleoptera | Scarabaeidae | *Rhabdotis buchardi* | Africa (Burkina Faso, Benin) | S. America (Brazil) |  |  |
| Coleoptera | Scarabaeidae | *Oryctes rhinoceros* (L.) | Asia (China, Thailand, Myanmar, Philippines, India) | Oceania (Solomon Islands) |  |  |
| Coleoptera | Scarabaeidae | *Xylotrupes gideon* (L.) | Asia (Thailand, Myanmar, India, Malaysia, Sabah, Thailand, Indonesia) | Oceania (P.N.G.) |  |  |
| Coleoptera | Tenebrionidae | *Tenebrio molitor* (L.) | Asia (China, Laos) | N. America (Mexico) |  |  |
| Coleoptera | Cerambycidae | *Ancylonotus tribulus* F.) | Africa (Senegal, Gabon) | S. America (South America) |  |  |
| Coleoptera | Curculionidae | *Rhynchophorus ferrugineus* (Oliv.) | Africa (DRC, Benin) | Oceania (P.N.G.) | Asia (Thailand, China) |  |
| Coleoptera | Curculionidae | *Rhynchophorus palmarum* (L.) | Africa (Uganda, Ivory Coast, Guinea) | S. America (pan-regional Barbados, Trinidad, Brazil, Ecuador, Venezuela) | N. America (West Indies, Mexico) |  |
| Coleoptera | Dynastidae | *Heteroligus meles* (Billberg) | Africa (Nigeria) | S. America (Brazil) |  |  |
| Coleoptera | Scarabaeidae Trichiinae | *Goliathus goliathus (*Zucht*.*) | Africa (DRC, Cameroon, CA Republic) | N. America (Mexico) |  |  |
| Dictyoptera | Blattidae | *Periplaneta americana* (L.) | Asia (China) | S. America (Brazil) | N. America (Mexico) |  |
| Dictyoptera | Blattidae | *Periplaneta australasiae* (F.) | Asia (China) | N. America (Mexico) |  |  |
| Dictyoptera | Ectobiidae | *Blattella germanica* (L.) | Asia (Japan) | N. America (Mexico) |  |  |
| Dictyoptera | Mantidae | *Tenodera* sp. | Asia (Malaysia, Sabah) | Oceania (P.N.G.) |  |  |
| Diptera | Muscidae | *Musca domestica* (L.) | Asia (China) | Africa (Ghana) | S. America (Mexico) |  |
| Hemiptera | Nepidae | *Nepa* sp. | Asia (Thailand) | Africa (Madagascar) |  |  |
| Hemiptera | Pentatomidae | *Nezara viridula* (L.) | Asia (India, Malaysia, Sabah, Indonesia, Kalimantan) | Africa (Nigeria) | Oceania (Papua) |  |
| Hemiptera/ Homoptera | Cicadidae | *Cosmopsaltria* sp. | Asia (Thailand) | Oceania (Papua) |  |  |
| Hemiptera/Homoptera | Dactylopiidae | *Dactylopius coccus* (Costa) | Asia (Canary Islands) | N. America (Mexico) |  |  |
| Heteroptera | Belostomatidae | *Belostoma* sp. | Africa (Congo) | S. America (Mexico) |  |  |
| Homoptera | Psyllidae | *Psylla* sp. | Africa (South Africa) | Au (Australia) |  |  |
| Hymenoptera | Apidae | *Bombus* sp. | Asia (Indonesia) | S. America (Mexico) |  |  |
| Hymenoptera | Apidae | *Trigona* sp. | Asia (Indonesia) | Au (Australia) | S. America (Mexico) |  |
| Hymenoptera | Apidae | *Xylocopa* sp. | Asia (Sri Lanka, Malaysia, Sabah, Indonesia, Kalimantan, India) | S. America (Mexico) |  |  |
| Hymenoptera | Formicidae | *Camponotus* sp. | Asia (Philippines, Indonesia) | N. America (Mexico) | Africa (Botswana) |  |
| Hymenoptera | Formicidae | *Oecophylla smaragdina* (F.) | Asia (China, Thailand, Myanmar, India) | Au (Australia) |  |  |
| Hymenoptera | Formicidae | *Oecophylla longinoda* (Latreille) | Africa (D.R. Congo) | S. America (Brazil) |  |  |
| Hymenoptera | Vespidae | *Eumenes* sp. | Asia (India) | N. America (Mexico) |  |  |
| Hymenoptera | Vespidae | *Polistes* sp. | Asia (Japan, India) | S. America (Guatemala) | N. America (Mexico) |  |
| Hymenoptera | Formicidae | *Carebara lignata* (Westwood) | Asia (China) | Africa (Zambia, South Africa, Zimbabwe, Botswana, Sudan, Mozambique, Namibia) |  |  |
| Isoptera | Termitidae | *Macrotermes* sp. | Asia (Indonesia, India) | Africa (DRC, Tanzania, Zimbabwe, Malawi, Zambia) | S. America (Colombia) |  |
| Isoptera | Termitidae | *Termes destructor* (DeGeer) | Asia (Indonesia) | S. America (Guyana) |  |  |
| Isoptera | Termitidae | *Termes fatalis* (L.) | Asia (Indonesia) | S. America (Guyana) |  |  |
| Lepidoptera | Noctuidae | *Agrotis infusa* (Boisd.) | Oceania (Australia) | Africa (Nigeria) |  |  |
| Lepidoptera | Bombycidae | *Bombyx mori* (L.) | Asia (Thailand, Myanmar) | N. America (Mexico) |  |  |
| Lepidoptera | Lasiocampidae | *Malacosoma* sp*.* | Asia (India) | N. America (N. Am.) |  |  |
| Lepidoptera | Nymphalidae | *Charaxes* spp | Africa (DRC) | N. America (Mexico) |  |  |
| Lepidoptera | Sphingidae | *Agrius convolvuli* (L.) | Asia (Indonesia, Japan) | Africa (Botswana, South Africa, Zambia) |  |  |
| Lepidoptera | Sphingidae | *Hippotion celerio* (L.) | Asia (Malaysia, Sabah) | Africa (Botswana) |  |  |
| Odonata | Acrididae | *Acrida turrita* (L.) | Asia (Korea) | Africa (Cameroon) |  |  |
| Odonata | Acrididae | *Locusta migratoria* (L.) | Asia (Thailand, China) | Oceania (P.N.G.) |  |  |
| Odonata | Acrididae | *Nomadacris septemfasciata* (Serville) | Asia (Kuwait, Saudi Arabia) | Africa (Congo, South Africa, Tanzania, Zambia, Zimbabwe, Malawi) |  |  |
| Odonata | Acrididae | *Schistocerca gregaria* (Forskål) | Asia (Pan-regional S.W. Asia, India) | Africa (Congo, Cameroon, Tanzania, Zambia, Uganda, South Africa, Botswana, Sudan, Ethiopia, Morocco) |  |  |
| Odonata | Aeshnidae | *Aeschna* sp. | Asia (India, Thailand) | N. America (Mexico) |  |  |
| Odonata | Aeshnidae | *Anax* sp. | Asia (Indonesia) | N. America (Mexico) |  |  |
| Orthoptera | Acrididae | *Acrida acuminata* (Stål) | S. America (Ecuador) | Africa (South Africa, Botswana, Malawi) |  |  |
| Orthoptera | Acrididae | *Catantops humeralis* (Thunberg) | Africa (Lesotho) | S. America (Brazil) |  |  |
| Orthoptera | Gryllidae | *Gryllus assimilis* (F.) | Africa (Malawi) | N. America (Mexico) |  |  |
| Orthoptera | Gryllidae | *Stenopelmatus* sp. | Africa (Malawi) | N. America (Mexico) |  |  |
| Orthoptera | Gryllotalpidae | *Gryllotalpa africana* (Palisot de Beauvois) | Asia (Japan, Thailand, Philippines, Vietnam, India, Indonesia, Laos) | Africa (Uganda, Zimbabwe) |  |  |
| Orthoptera | Tettigoniidae | *Gymnoproctus sculpturatus* (Karsch) | Africa (Benin) | S. America (Brazil) |  |  |
| Orthoptera | Acrididae | *Cyrtacanthacris tatarica* (L.) | Asia (Thailand, Indonesia) | Africa (Botswana, Zambia) |  |  |
| Orthoptera | Acrididae | *Schistocerca* sp. | Asia (India) | S. America (Ecuador, Argentina) | Africa (S. Africa) | N. America (Mexico) |
| Orthoptera | Acrididae | *Truxaloides braziliensis* (Drury) | Africa (Lesotho) | South America (Brazil) |  |  |
| Orthoptera | Acrididae | *Truxaloides constrictus* (Thunberg) | Africa (Zimbabwe) | South America (Brazil) |  |  |
| Orthoptera | Gryllidae | *Gryllus bimaculatus* (De Geer) | Asia (China, Thailand, India, Laos) | Africa (Zambia, G. Bissau, Sierra Leone, Guinea, Liberia, Benin, Togo, Nigeria, D.R. Congo, Uganda, Kenya) |  |  |

**Supplementary Table 2**

Number of potentially edible insect species, reported edible insect species and proportion of consumed insect insects per country

| **Continent** | **Country/ territory** | **All potentially edible insect species present** | **Reported edible insect species** | **Proportion of consumed insect species** | **Continent** | **Country/ territory** | **All potentially edible insect species present** | **Reported edible insect species** | **Proportion of consumed insect species** | **Continent** | **Country/ territory** | **All potentially edible insect species present** | **Reported edible insect species** | **Proportion of consumed insect species** | **Continent** | **Country/ territory** | **All potentially edible insect species present** | **Reported edible insect species** | **Proportion of consumed insect species** |
| --- | --- | --- | --- | --- | --- | --- | --- | --- | --- | --- | --- | --- | --- | --- | --- | --- | --- | --- | --- |
| Africa | DRC | 277 | 255 | 92.1 | Asia | Thailand | 285 | 272 | 95.4 | Oceania | Papua New Guinea | 50 | 42 | 84.0 | N. America | USA | 27 | 6 | 22.2 |
| Africa | Cameroon | 109 | 100 | 91.7 | Asia | India | 281 | 263 | 93.6 | Oceania | New Zealand | 14 | 4 | 28.6 | N. America | Canada | 10 | 4 | 40.0 |
| Africa | Zambia | 90 | 78 | 86.7 | Asia | China | 270 | 235 | 87.0 | Oceania | New Caledonia | 13 | 4 | 30.8 | N. America | Dominica | 9 | 0 | 0.0 |
| Africa | South Africa | 79 | 56 | 70.9 | Asia | Japan | 138 | 123 | 89.1 | Oceania | Solomon Isl. | 6 | 1 | 16.7 | N. America | Guadeloupe | 9 | 0 | 0.0 |
| Africa | Zimbabwe | 69 | 52 | 75.4 | Asia | Indonesia | 107 | 88 | 82.2 | Oceania | Australia | 78 | 62 | 79.5 | N. America | Jamaica | 9 | 0 | 0.0 |
| Africa | CR Africa | 68 | 62 | 91.2 | Asia | Malaysia | 80 | 65 | 81.3 | Oceania | Austria | 10 | 0 | 0.0 | N. America | Costa Rica | 8 | 0 | 0.0 |
| Africa | Botswana | 53 | 43 | 81.1 | Asia | Lao PDR | 56 | 50 | 89.3 | Oceania | Norfolk Island | 3 | 0 | 0.0 | N. America | Martinique | 8 | 0 | 0.0 |
| Africa | Madagascar | 48 | 31 | 64.6 | Asia | Viet Nam | 34 | 20 | 58.8 | Europe | France | 27 | 0 | 0.0 | N. America | Belize | 6 | 0 | 0.0 |
| Africa | Benin | 43 | 27 | 62.8 | Asia | Philippines | 32 | 18 | 56.3 | Europe | Spain | 20 | 0 | 0.0 | N. America | Bermuda | 6 | 0 | 0.0 |
| Africa | Malawi | 39 | 28 | 71.8 | Asia | South Korea | 23 | 10 | 43.5 | Europe | Portugal | 19 | 0 | 0.0 | N. America | Cuba | 6 | 0 | 0.0 |
| Africa | Nigeria | 37 | 25 | 67.6 | Asia | Myanmar | 23 | 14 | 60.9 | Europe | UK | 17 | 0 | 0.0 | N. America | Puerto Rico | 6 | 0 | 0.0 |
| Africa | Kenya | 35 | 18 | 51.4 | Asia | Iran | 22 | 13 | 59.1 | Europe | Greece | 16 | 0 | 0.0 | N. America | British Virgin Isl. | 5 | 0 | 0.0 |
| Africa | Tanzania | 35 | 21 | 60.0 | Asia | Türkiye | 21 | 6 | 28.6 | Europe | Italy | 16 | 0 | 0.0 | N. America | El Salvador | 4 | 0 | 0.0 |
| Africa | Namibia | 31 | 15 | 48.4 | Asia | Cambodia | 15 | 4 | 26.7 | Europe | Germany | 15 | 0 | 0.0 | N. America | Bahamas | 3 | 0 | 0.0 |
| Africa | Mozambique | 27 | 12 | 44.4 | Asia | Sri Lanka | 15 | 4 | 26.7 | Europe | Netherlands | 14 | 0 | 0.0 | N. America | Cayman Islands | 3 | 0 | 0.0 |
| Africa | Sao Tome and Principe | 22 | 6 | 27.3 | Asia | Singapore | 13 | 1 | 7.7 | Europe | Belgium | 12 | 0 | 0.0 | N. America | Antigua and Barbuda | 2 | 0 | 0.0 |
| Africa | Uganda | 22 | 11 | 50.0 | Asia | Hong Kong | 12 | 0 | 0.0 | Europe | Sweden | 11 | 0 | 0.0 | N. America | Anguilla | 2 | 0 | 0.0 |
| Africa | Niger | 22 | 17 | 77.3 | Asia | Israel | 12 | 3 | 25.0 | Europe | Switzerland | 11 | 0 | 0.0 | N. America | Turks and Caicos Isl. | 3 | 0 | 0.0 |
| Africa | Burkina Faso | 22 | 19 | 86.4 | Asia | Russia | 11 | 0 | 0.0 | Europe | Denmark | 8 | 0 | 0.0 | N. America | Barbados | 2 | 0 | 0.0 |
| Africa | Gabon | 21 | 12 | 57.1 | Asia | Kuwait | 11 | 3 | 27.3 | Europe | Bulgaria | 9 | 0 | 0.0 | N. America | Haiti | 2 | 0 | 0.0 |
| Africa | Angola | 21 | 17 | 81.0 | Asia | Saudi Arabia | 10 | 3 | 30.0 | Europe | Croatia | 9 | 0 | 0.0 | N. America | Grenada | 1 | 0 | 0.0 |
| Africa | Senegal | 20 | 12 | 60.0 | Asia | Timor-Leste | 9 | 0 | 0.0 | Europe | Finland | 9 | 0 | 0.0 | N. America | West Indies | 1 | 1 | 100.0 |
| Africa | Ghana | 18 | 11 | 61.1 | Asia | Bangladesh | 9 | 1 | 11.1 | Europe | French Guiana | 9 | 0 | 0.0 | Oceania | Guam | 8 | 0 | 0.0 |
| Africa | Togo | 16 | 9 | 56.3 | Asia | United Arab Emirates | 9 | 1 | 11.1 | Europe | French S. Territories | 10 | 0 | 0.0 | Oceania | N. Mariana Islands | 7 | 0 | 0.0 |
| Africa | Morocco | 15 | 4 | 26.7 | Asia | Oman | 8 | 1 | 12.5 | Europe | Hungary | 8 | 0 | 0.0 | Oceania | Tonga | 7 | 0 | 0.0 |
| Africa | Burundi | 14 | 13 | 92.9 | Asia | Pakistan | 8 | 1 | 12.5 | Europe | Norway | 8 | 0 | 0.0 | Oceania | Fiji | 5 | 0 | 0.0 |
| Africa | Lesotho | 14 | 14 | 100 | Asia | Macau | 7 | 1 | 14.3 | Europe | Ukraine | 8 | 0 | 0.0 | Oceania | Palau | 5 | 0 | 0.0 |
| Africa | Equatorial Guinea | 8 | 5 | 62.5 | Asia | Cocos (Keeling Islands) | 2 | 1 | 50.0 | Europe | Bosnia and Herzegovina | 7 | 0 | 0.0 | Oceania | Wallis and Futuna | 4 | 0 | 0.0 |
| Africa | Egypt | 11 | 3 | 27.3 | Asia | Nepal | 7 | 4 | 57.1 | Europe | Czechia | 7 | 0 | 0.0 | Oceania | Cook Islands | 3 | 0 | 0.0 |
| Africa | South Sudan | 11 | 10 | 90.9 | Asia | Kalimantan | 7 | 7 | 100.0 | Europe | Estonia | 7 | 0 | 0.0 | Oceania | Micronesia | 3 | 0 | 0.0 |
| Africa | Ethiopia | 10 | 1 | 10.0 | Asia | Bhutan | 6 | 1 | 16.7 | Europe | Poland | 7 | 0 | 0.0 | Oceania | American Samoa | 2 | 0 | 0.0 |
| Africa | Côte d’Ivoire | 10 | 3 | 30.0 | Asia | Lebanon | 6 | 1 | 16.7 | Europe | Romania | 7 | 0 | 0.0 | Oceania | Marshall Islands | 2 | 0 | 0.0 |
| Africa | Guinea | 10 | 4 | 40.0 | Asia | Bahrain | 6 | 2 | 33.3 | Europe | Albania | 6 | 0 | 0.0 | Oceania | Kiribati | 1 | 0 | 0.0 |
| Africa | Algeria | 9 | 1 | 11.1 | Asia | Syria | 6 | 4 | 66.7 | Europe | Honduras | 6 | 0 | 0.0 | S. America | Mexico | 508 | 450 | 88.6 |
| Africa | Mauritius | 9 | 2 | 22.2 | Asia | Kazakhstan | 5 | 0 | 0.0 | Europe | Luxembourg | 6 | 0 | 0.0 | S. America | Brazil | 141 | 140 | 99.3 |
| Africa | Tunisia | 9 | 2 | 22.2 | Asia | Christmas Isl. | 5 | 1 | 20.0 | Europe | Montenegro | 6 | 0 | 0.0 | S. America | Ecuador | 106 | 93 | 87.7 |
| Africa | Cape Verde | 8 | 0 | 0.0 | Asia | Jordan | 5 | 2 | 40.0 | Europe | Georgia | 5 | 0 | 0.0 | S. America | Colombia | 76 | 62 | 81.6 |
| Africa | Gambia | 8 | 0 | 0.0 | Asia | Palestine | 4 | 1 | 25.0 | Europe | Ireland | 5 | 0 | 0.0 | S. America | Venezuela | 55 | 49 | 89.1 |
| Africa | Réunion | 8 | 0 | 0.0 | Asia | Afghanistan | 4 | 2 | 50.0 | Europe | Lithuania | 5 | 0 | 0.0 | S. America | Argentina | 22 | 17 | 77.3 |
| Africa | Sierra Leone | 8 | 4 | 50.0 | Asia | Sinai Desert | 4 | 4 | 100 | Europe | Malta | 5 | 0 | 0.0 | S. America | Peru | 18 | 8 | 44.4 |
| Africa | Sudan | 13 | 7 | 53.8 | Asia | Azerbaijan | 3 | 0 | 0.0 | Europe | Serbia | 5 | 0 | 0.0 | S. America | Guyana | 11 | 6 | 54.5 |
| Africa | Chad | 6 | 2 | 33.3 | Asia | Brunei | 3 | 1 | 33.3 | Europe | Slovenia | 5 | 0 | 0.0 | S. America | Panama | 10 | 2 | 20.0 |
| Africa | Seychelles | 5 | 0 | 0.0 | Asia | Kyrgyzstan | 3 | 1 | 33.3 | Europe | Cyprus | 4 | 0 | 0.0 | S. America | Paraguay | 10 | 3 | 30.0 |
| Africa | Eritrea | 4 | 1 | 25.0 | Asia | Maldives | 3 | 1 | 33.3 | Europe | Slovakia | 4 | 0 | 0.0 | S. America | Bolivia | 9 | 3 | 33.3 |
| Africa | Mayotte | 4 | 0 | 0.0 | Asia | Taiwan | 3 | 3 | 100 | Europe | Belarus | 3 | 0 | 0.0 | S. America | Nicaragua | 9 | 4 | 44.4 |
| Africa | Swaziland | 4 | 0 | 0.0 | Asia | Armenia | 2 | 1 | 50.0 | Europe | Gibraltar | 2 | 0 | 0.0 | S. America | Guatemala | 8 | 2 | 25.0 |
| Africa | G. Bissau | 4 | 0 | 0.0 | Asia | Qatar | 7 | 1 | 14.3 | Europe | Moldova | 2 | 0 | 0.0 | S. America | Chile | 8 | 3 | 37.5 |
| Africa | Liberia | 4 | 1 | 25.0 | Asia | Turkmenistan | 2 | 1 | 50.0 | Europe | Montserrat | 2 | 0 | 0.0 | S. America | Trinidad and Tobago | 6 | 0 | 0.0 |
| Africa | Mauritania | 4 | 1 | 25.0 | Asia | Uzbekistan | 2 | 1 | 50.0 | Europe | Andorra | 1 | 0 | 0.0 | S. America | Suriname | 6 | 1 | 16.7 |
| Africa | Somalia | 4 | 1 | 25.0 | Asia | Yemen | 2 | 2 | 100 | Europe | Guernsey | 1 | 0 | 0.0 | S. America | Uruguay | 6 | 1 | 16.7 |
| Africa | Mali | 4 | 2 | 50.0 | Asia | Canary Islands | 1 | 1 | 100 | Europe | Isle of Man | 1 | 0 | 0.0 | S. America | Aruba | 1 | 0 | 0.0 |
| Africa | Libya | 2 | 1 | 50.0 | Asia | Mongolia | 1 | 1 | 100 | Europe | Kosovo | 1 | 0 | 0.0 |  |  |  |  |  |
| Africa | Rwanda | 2 | 2 | 100 | Asia | North Korea | 1 | 1 | 100 | Europe | Latvia | 1 | 0 | 0.0 |  |  |  |  |  |
| Africa | Comoros | 1 | 0 | 0.0 | Asia | Tajikistan | 1 | 1 | 100 | Europe | Liechtenstein | 1 | 0 | 0.0 |  |  |  |  |  |
| Africa | Djibouti | 1 | 0 | 0.0 | Asia |  |  |  |  |  |  |  |  |  |  |  |  |  |  |
